# Mutational landscape differences between young-onset and older-onset breast cancer patients

**DOI:** 10.1186/s12885-020-6684-z

**Published:** 2020-03-12

**Authors:** Nicole E. Mealey, Dylan E. O’Sullivan, Joy Pader, Yibing Ruan, Edwin Wang, May Lynn Quan, Darren R. Brenner

**Affiliations:** 1 Department of Oncology, Cumming School of Medicine, University of Calgary, Calgary, Alberta Canada; 2 Department of Public Health Sciences, Queen’s University, Kingston, Ontario Canada; 3 Department of Cancer Epidemiology and Prevention Research, CancerControl Alberta, Alberta Health Services, Calgary, Alberta Canada; 4 Department of Biochemistry & Molecular Biology, Cumming School of Medicine, University of Calgary, Calgary, Alberta Canada; 5 Department of Community Health Sciences, Cumming School of Medicine, University of Calgary, Calgary, Alberta Canada; 6 Department of Surgery, Cumming School of Medicine, University of Calgary, Calgary, Alberta Canada

**Keywords:** Mutational signatures, Breast cancer, Young women, Genomics, Somatic mutations

## Abstract

**Background:**

The incidence of breast cancer among young women (aged ≤40 years) has increased in North America and Europe. Fewer than 10% of cases among young women are attributable to inherited *BRCA1* or *BRCA2* mutations, suggesting an important role for somatic mutations. This study investigated genomic differences between young- and older-onset breast tumours.

**Methods:**

In this study we characterized the mutational landscape of 89 young-onset breast tumours (≤40 years) and examined differences with 949 older-onset tumours (> 40 years) using data from The Cancer Genome Atlas. We examined mutated genes, mutational load, and types of mutations. We used complementary R packages “deconstructSigs” and “SomaticSignatures” to extract mutational signatures. A recursively partitioned mixture model was used to identify whether combinations of mutational signatures were related to age of onset.

**Results:**

Older patients had a higher proportion of mutations in *PIK3CA*, *CDH1*, and *MAP3K1* genes, while young-onset patients had a higher proportion of mutations in *GATA3* and *CTNNB1*. Mutational load was lower for young-onset tumours, and a higher proportion of these mutations were C > A mutations, but a lower proportion were C > T mutations compared to older-onset tumours. The most common mutational signatures identified in both age groups were signatures 1 and 3 from the COSMIC database. Signatures resembling COSMIC signatures 2 and 13 were observed among both age groups. We identified a class of tumours with a unique combination of signatures that may be associated with young age of onset.

**Conclusions:**

The results of this exploratory study provide some evidence that the mutational landscape and mutational signatures among young-onset breast cancer are different from those of older-onset patients. The characterization of young-onset tumours could provide clues to their etiology which may inform future prevention. Further studies are required to confirm our findings.

## Background

### Young-onset breast cancer

Breast cancer is the most common non-keratinocyte cancer among women. Its estimated global incidence was 1.68 million in 2012, accounting for 25% of cancer diagnoses among women of all ages [1]. Approximately 7% of all breast cancer diagnoses occur before age 40 [2–4]. Among women under the age of 40 in the United States, breast cancer accounts for over 40% of cancer diagnoses [2]. Epidemiological studies demonstrated a trend of rising incidence among Canadian women between 1969 and 2012, and among women in the United States from 2004 to 2015 [5, 6]. A European study showed an average increase of 3 and 1% annually among women aged 20–29, and 30–39, respectively [3].

Young age at diagnosis is generally defined as a diagnosis before age 40 [2, 7], but can be stratified by other ages. Current Canadian screening guidelines do not recommend screening for breast cancer until age 50, unless the patient is at increased risk for breast cancer [8]. Young age at diagnosis is linked to later stage of tumour progression at diagnosis, and worse clinical outcomes [2, 7, 9–14]. Breast tumours among young women are more likely to have histological markers of worse prognosis, including large size, poor differentiation, higher grade, and vascular or lymphatic invasion [2, 7, 12]. Of the four well defined subtypes of breast cancer (human epidermal growth factor receptor 2 over-expressing (HER2+), triple-negative breast cancer (TNBC), luminal A, and luminal B), cancers diagnosed among young women are more likely to be TNBC or HER2+ [2, 14, 15]. These tumour types are linked to shorter survival [2, 14, 15]. Previous analyses among young-onset breast cancer patients reported 26% of tumours being TNBC compared to only 12% in overall populations [14]. An alternative molecular categorization often used for classification of breast tumours (prediction analysis of microarrays 50 gene set, PAM50) includes basal-like, luminal A, luminal B, HER2+, and normal-like groups [16]. Of these categories, young women were more likely to be diagnosed with the more aggressive basal-like and HER2+ tumour types [16]. Liao et al. observed a stronger inverse association between ER expression and age than between ER expression and menopausal status [13]. This result suggests that age may be the more biologically relevant metric to stratify breast cancer patients for examining genomic differences.

Survival rates for breast cancer patients younger than 40 years are lower than those for women 40 or older [2, 14]. This trend holds even when controlling for histological subtype, disease stage, and other prognostic features. Within the luminal A, luminal B, and TNBC subtypes, young age is associated with worse breast cancer-specific survival, and there was no significant association by age among HER2+ tumours [17]. A large retrospective study showed that disease-specific mortality was 39% higher among patients under 40 years compared to that of patients 40 or over (95% CI 35–45%) [7]. Anders et al. observed that patients under age 40 had worse disease free survival compared to the 40–45 age group, although it was consistent among 5-year strata under age 40 [15]. Young breast cancer patients have a higher risk of both local and distal recurrence [2, 14, 18]. One study found an increased risk for local recurrence among younger women, for both women under 35 years compared to over 50 (Hazard Ratio (HR) = 2.80 (95% CI 1.41–5.60)) and women 35–50 years compared to over 50 (HR = 1.72 (95% CI 1.17–2.54)) [19]. Another study showed that the 5-year local recurrence free survival rate for breast cancer patients under 40 years was significantly lower than that of patients 40 and over [20].

Breast tumours among young women have more aggressive histologic and molecular markers. Young age is associated with lower survival even when controlling for tumour subtype. Young women diagnosed with breast cancer have a higher risk of recurrence and more severe psychosocial consequences such as concerns over premature menopause. These factors demonstrate the importance of understanding young-onset breast cancer, despite its lower incidence relative to older-onset breast cancer.

### Somatic mutations

Less than 10% of breast cancer incidence among young women is attributable to heritable mutations in the *BRCA1* or *BRCA2* genes. Further, Bryan et al. (2017) found no evidence that germline mutations are related to mortality or tumour aggressiveness among breast cancer patients under age 50 [21]. Neither the incidence nor worse outcomes seen in young breast cancer patients can be entirely explained by inherited mutations. This suggests an important role for somatic mutations caused by lifestyle or environmental exposures in combination with intrinsic processes in tumour initiation and development.

Patterns in these somatic mutations can be examined using mutational signatures. This approach considers Single Nucleotide Variants (SNVs) from next generation sequencing of whole exomes (WES) in their 3-nucleotide context. The array of mutation types is represented in a mutational spectrum, then decomposed into recurring patterns, referred to as mutational signatures. Thirty validated mutational signatures are listed in the Catalogue Of Somatic Mutations In Cancer (COSMIC).

We analyzed somatic mutations in breast tumours to investigate whether there are differences between young- and older-onset breast cancer patients to better understand the unique genetic characteristics of young-onset breast cancers. In addition, we sought to explore unique mutational signatures by age groups as an exploratory analyses for insights into potential differences in etiology among young-onset patients. No previous research has investigated differences in mutational signatures found in breast tumours stratified by age.

## Methods

### Data

Both clinical and genomic data were obtained from The Cancer Genome Atlas (TCGA) Breast Invasive Carcinoma project (TCGA-BRCA, dbGaP study accession = phs000178) [5]. Clinical data files, and simple somatic mutation files in Variant Call Format (VCF) were obtained for all 1044 cases where simple somatic mutation data were available. WES had a minimum of 70% coverage at 20x depth [22]. VCF files were based on WES and produced via the MuTect2 workflow [23]. These files were downloaded using the Genomic Data Commons Data Transfer Tool [24] on June 26, 2018. PAM50 tumour subtypes were retrieved from cBioPortal [22] on January 17, 2019 and from Supplementary Table 1 of a paper by Ciriello et al. [25].

Clinical files were sorted by the patient age listed in them, and then matched with the corresponding mutation files from the same patient. An age cut-off of 40 years was used to divide patients into young and older age groups, as used previously in literature [2, 20]. For a series of our analyses we also compared patients 40 years of age and younger to patients over 60 years of age. Cases were only included if there were both somatic mutation and clinical data available, including the patient’s age. For tumour type analyses, cases without available PAM50 tumour type data were excluded. TCGA included some breast cancer patients with multiple VCF files, indicating that more than one tumour sample was submitted for the same patient, possibly from different parts of the tumour which may be heterogeneous. Analyses included only the first VCF file listed in the downloaded directory. Somatic mutations in the VCF files were filtered to remove any variants that did not pass all quality filters applied by the MuTect2 algorithm, and insertions and deletions, leaving only high quality SNVs.

Having a sufficiently high number of mutations is of particular importance for identifying flat mutational signatures [26]. Samples with a low number of mutations tend to have a higher sum of squared errors of prediction between the original and calculated mutational spectra [26]. Low mutation number may also lead to overfitting, and the identification of spurious signatures. Therefore, cases were excluded from the mutation type and mutational signatures analyses if they contained too few mutations, defined in this study as fewer than 40 mutations [27]. These cases were retained for the analyses of mutated genes and mutational load.

### Mutated genes, mutational load and mutation types

We aimed to determine which genes showed differential mutation prevalence between young- and older-onset breast cancer cases. Portions of our code were adapted from a guide for mapping loci of SNPs to genes in R (Version 1.1.453) [28]. Gene loci were identified using the GDC.h38 GENCODE v22 GTF file, which corresponds to the reference genome used by TCGA [28]. The “*findOverlaps”* function from the *“GenomicRanges”* package was used to compare SNVs to gene loci to find mutations within genes [29]. The genes we selected for study were based on the list of 46 genes that Berger et al. found to be significantly mutated among gynecologic and breast cancers using TCGA data [30]. We reduced the risk for false discovery by limiting the number of genes we investigated to this set of genes which are likely relevant to breast cancer. For each gene the number of tumour samples with at least one mutation in the gene was counted. A Fisher’s exact test was used to identify differences in the proportion of mutated samples in each gene between the young and older, and between the young and oldest age groups. We chose not to adjust for multiple comparisons because this study is exploratory in nature, and the relatively small sample size of the young-onset group makes multiple comparisons of less concern.

We observed the total number of somatic mutations in each tumour sample, and compared the distribution of mutational loads between the age groups. A Welch Two Sample t-test was conducted in R to test for a difference between the log-transformed mutational loads of the two age groups. Given that SNVs comprise a large proportion of somatic mutations, we also reported the SNV-only mutational load, and tested difference by age groups.

We examined the number of each type of SNV (i.e. C > A, C > G, C > T, T > A, T > C, T > G) among young and older breast cancer patients, and calculated the proportion made up by each type. The proportion of each type between the age groups overall was compared using Welch’s t-test.

### Mutational signatures

The mutational signatures of young and older breast cancer patients’ tumours were investigated using two R packages with complementary approaches to determining signatures:“deconstructSigs” [26], and “SomaticSignatures” [31]. The first, “deconstructSigs,” uses an iterative approach to calculate the combination of COSMIC signatures that best approximate a tumour’s mutational spectrum. “SomaticSignatures” takes a cohort of tumours’ mutational spectra and uses either principal component analysis or Non-negative Matrix Factorization (NMF) to identify signatures that are present within the cohort, and their contribution to each tumour’s mutational spectrum. Consequently, “deconstructSigs” can analyze individual samples and the results are more comparable to previous studies, while “SomaticSignatures” requires multiple samples but may identify novel signatures among them. We employed both packages in order to examine whether a data driven approach in “SomaticSignatures” would suggest signatures in younger patients in contrast to a confirmatory approach using deconstructSigs from the COSMIC repository of signatures.

#### deconstructSigs

Within the “deconstructSigs” package, the “mut.to.sigs.input” method was used to construct the appropriate input data structure. Then we used “whichSignatures” to determine which of the COSMIC signatures were present in the tumour samples and their contribution towards the total mutational spectra [26]. This function uses an iterative algorithm to find the combination and relative weight of signatures that best matches each mutational spectrum. The GRCh38 reference genome was used for this study. We calculated the mean contribution of signatures across samples from the young-onset and older-onset groups. We also wanted to determine whether there was a difference in prevalence of certain signatures by age of diagnosis. A chi-square test with adjustment for the false discovery rate between age groups was used to test for a difference in the proportion of tumours with each signature present above a threshold of 6%. The 6% threshold is a convention integrated into “deconstructSigs” [26].

#### SomaticSignatures

The 3-nucleotide mutational context of each SNV was extracted using the “mutationContext” method from the “SomaticSignatures” package. This function compares the locus of each SNV to the corresponding reference genome GRCh38.d1.vd1 to identify the nucleotides immediately 3′ and 5′ of the SNV. Next, the frequencies of each of the 96 alteration types was calculated with the “motifMatrix” method of the same package. To determine how many signatures we expect to identify we ran “assessNumberSignatures”, and saw reduced improvements to RSS and explained variance when incrementing the number of signatures by one above five signatures in both age groups. Computing mutational signatures using the “deconstructSigs” package identified sixteen COSMIC signatures with a mean contribution over 1% among young cases. Therefore, we chose to search for five and sixteen signatures separately in each age group. The “identifySignatures” method was used to decompose the mutational spectra of individuals in each age group into novel signatures, using the NMF option.

### Tumour subtype

We examined the proportion of tumours in each PAM50 subtype within young- and older-onset groups. Tumour type was linked with age group or presence of mutational signatures using patient barcodes. There were 852 cases (78 young and 774 older, including 372 diagnosed > 60 years) with tumour type data available. We tested for differences in proportions of tumour types across age groups using Fisher’s exact test. We also used Fisher’s exact test to examine differences in proportions of tumour types with each signature present. There is a high potential for error when identifying signatures in samples with very low mutational burdens. For this reason, cases with fewer than 40 mutations were excluded in the test of signature prevalence for each tumour type, leaving 848 cases (77 young and 771 older, including 372 diagnosed > 60 years) with tumour type data.

### Hierarchical clustering analysis

To determine if specific combinations of mutational signature contributions were related to young-onset breast cancer tumours, we employed a Recursively Partitioned Mixture Model (RPMM) clustering analysis using R package “RPMM” [32] on the mutational signatures that had the greatest variability, based on the results from “deconstructSigs”. The mutational signatures were determined to have adequate variability if the signature had an interquartile range (IQR) above 0% or a standard deviation greater than 10%. In order for the signatures to be on the same scale, each signature was normalized before the RPMM was conducted.

Given that mutational signature clusters may vary by breast cancer subtype and that these have been shown to be distributed disproportionately by age, we restricted this analysis to only subjects that had intrinsic subtype data determined by the PAM50 classification (*n* = 848). From this classification, five tumour subtypes were used: basal-like, HER2+, luminal A, luminal B, and normal-like [33].

To determine the unadjusted relationship of mutational signature combination class membership with age and PAM50 subtype we employed chi-square permutation tests running 10,000 permutations. To determine the relationship of mutational signature combinations and class membership for age and PAM50 subtype that were mutually adjusted, we conducted logistic regression models with the outcome being the class of interest versus all other classes.

## Results

Of the 1098 breast cancer cases available from TCGA, 1097 had clinical data, and 1044 cases had data on simple somatic mutations. Including the multiple somatic mutation files available for some cases, there were 1097 clinical files and 1080 somatic mutation files downloaded. After removing redundant samples for the same patient (*n* = 37), there were 952 older and 91 young samples remaining. For the analyses of mutation type and mutational signatures, samples that contained fewer than 40 SNVs (*n* = 5) were removed, leaving 949 older and 89 young samples. PAM50 data was available for 78 and 774 young- and older-onset cases, respectively, and these cases were included when testing for a difference in tumour types by age. After removing samples with an insufficient number of SNVs, there were 77 young-onset tumours and 771 older-onset tumours with available PAM50 categories. These were used for investigating differences in tumour types by mutational signature.

### Tumour subtype

We examined the PAM50 subtypes of tumours in each age group (Table 1). Approximately half of cases were in the luminal A subtype in both age groups, and almost a quarter were luminal B. The basal-like subtype accounted for 22% of the young-onset cases and 16.7% of the older-onset cases. The HER2+ and normal-like subtypes were the least common, accounting for approximately 8 and 3% of cases overall, respectively. There were no statistically significant differences in proportion of tumour subtype when comparing the ≤40 to > 40 age groups. However, we also compared young cases (≤40 years) to cases diagnosed at > 60 years of age. In this comparison, we found that the basal-like subtype was significantly more common among young patients than among patients diagnosed after age 60. There was low power for the normal-like and HER2+ subtypes.

**Table 1:** Tumour subtypes across age of onset groups

Tumour Type	Young	Older	Oldest	Total	p-value (young vs. older)	p-value (young vs. oldest)
Basal-like	17 (22%)	129 (16.7%)	45 (12%)	146 (17.1%)	0.27	0.03*
HER2+	3 (4%)	66 (8.5%)	27 (7%)	69 (8.1%)	0.19	0.33
Luminal A	39 (50%)	393 (50.8%)	219 (59%)	432 (50.7%)	0.91	0.17
Luminal B	18 (23%)	164 (21.2%)	75 (20%)	182 (21.4%)	0.67	0.54
Normal-like	1 (1%)	22 (2.8%)	6 (2%)	23 (2.7%)	0.71	1.00

Frequency and proportion of tumours in each age of onset group of each PAM50 tumour subtype. Entries are the number of samples of each subtype (% of samples in age of onset group). The *p*-values were calculated using Fisher’s exact test (* indicates statistical significance at the 0.05 level). Age of onset groups were defined as “Young”: breast tumours diagnosed at ≤40 years of age (*n* = 78), “Older”: breast tumours diagnosed > 40 years of age (*n* = 774), and “Oldest”: breast tumours diagnosed > 60 years of age (*n* = 372). HER2+: human epidermal growth factor receptor 2 over-expressing; PAM50: prediction analysis of microarrays 50 gene set

We also examined the proportion of samples with each signature present across each tumour type (Table 2, Additional file 1: Figure S1). Signatures 1 and 3 were the most common signatures across all tumour types, with the exception of HER2+ tumours, in which signature 2 had a slightly higher prevalence than signature 3. Nine signatures showed a statistically significant difference across tumour types: signatures 2, 3, 5, 7, 13, 16, 18, 22, and 27. Signature 3 prevalence was elevated among basal-like tumours and signature 27 was most common among normal-like tumours, while signatures 2 and 13 were more common among HER2+ tumours. Signature 22 had higher prevalence among luminal A and normal-like tumours. HER2+, luminal A and luminal B tumours had a higher prevalence of signature 5, while HER2+, luminal A and normal-like tumours had a higher prevalence of signature 7. Signature 16 had a higher prevalence among luminal A and B tumours, but a lower prevalence among basal-like and HER2+ tumours. Signature 18 was most common among normal-like tumours, followed by luminal A and B tumours. The difference in prevalence between tumour types was consistent when divided by age group for some signatures (signatures 2 and 3). For others, it was only present among the older-onset groups (signatures 5, 7, 13, 16, 22, and 27), or only when age groups were combined (signature 18). Signatures 8, 24, and 29 only showed a difference between tumour types in the young-onset group, whereas signature 25 only showed a difference among tumours diagnosed after age 60. Signature 30 had opposing trends in the different age groups; it was more prevalent among basal-like and HER2+ young-onset tumours, but less prevalent among HER2+ older-onset tumours.

**Table 2:** Breast tumours with each COSMIC signature present across tumour subtypes

Signature	Age group	Basal-like	HER2+	Luminal A	Luminal B	Normal-like	p-value	Proposed Etiology
Signature 1	Total	107 (73%)	56 (81%)	330 (77%)	139 (76%)	14 (64%)	0.45	Spontaneous deamination of 5-methylcytosine
	*Young*	*14 (82%)*	*2 (67%)*	*28 (74%)*	*11 (61%)*	*1 (100%)*	*0.65*	
	*Older*	*93 (72%)*	*54 (82%)*	*302 (77%)*	*128 (78%)*	*13 (62%)*	*0.27*	
	*Oldest*	*36 (80%)*	*20 (74%)*	*175 (80%)*	*61 (81%)*	*4 (67%)*	*0.80*	
Signature 2	Total	28 (19%)	47 (68%)	169 (39%)	59 (32%)	8 (36%)	< 0.01*	AID/APOBEC
	*Young*	*3 (18%)*	*2 (67%)*	*14 (37%)*	*2 (11%)*	*1 (100%)*	0.04*	
	*Older*	*25 (19%)*	*45 (68%)*	*155 (40%)*	*57 (35%)*	*7 (33%)*	< 0.01*	
	*Oldest*	*12 (27%)*	*19 (70%)*	*82 (37%)*	*22 (29%)*	*3 (50%)*	< 0.01*	
Signature 3	Total	134 (92%)	46 (67%)	252 (59%)	136 (75%)	15 (68%)	< 0.01*	Defective DNA double-strand break repair by homologous recombination
	*Young*	*17 (100%)*	*1 (33%)*	*20 (53%)*	*14 (78%)*	*1 (100%)*	< 0.01*	
	*Older*	*117 (91%)*	*45 (68%)*	*232 (59%)*	*122 (74%)*	*14 (67%)*	< 0.01*	
	*Oldest*	*41 (91%)*	*18 (67%)*	*125 (57%)*	*55 (73%)*	*3 (50%)*	< 0.01*	
Signature 4	Total	26 (18%)	4 (6%)	48 (11%)	24 (13%)	3 (14%)	0.11	Smoking tobacco
	*Young*	*3 (18%)*	*1 (33%)*	*6 (16%)*	*5 (28%)*	*0 (0%)*	*0.60*	
	*Older*	*23 (18%)*	*3 (5%)*	*42 (11%)*	*19 (12%)*	*3 (14%)*	*0.07*	
	*Oldest*	*7 (16%)*	*2 (7%)*	*24 (11%)*	*8 (11%)*	*1 (17%)*	*0.75*	
Signature 5	Total	17 (12%)	18 (26%)	145 (34%)	50 (27%)	3 (14%)	< 0.01*	Unknown
	*Young*	*1 (6%)*	*0 (0%)*	*13 (34%)*	*7 (39%)*	*0 (0%)*	*0.08*	
	*Older*	*16 (12%)*	*18 (27%)*	*132 (34%)*	*43 (26%)*	*3 (14%)*	< 0.01*	
	*Oldest*	*4 (9%)*	*9 (33%)*	*82 (37%)*	*19 (25%)*	*2 (33%)*	< 0.01*	
Signature 6	Total	23 (16%)	7 (10%)	61 (14%)	25 (14%)	1 (5%)	0.64	Defective DNA mismatch repair
	*Young*	*3 (18%)*	*0 (0%)*	*8 (21%)*	*4 (22%)*	*0 (0%)*	*1.00*	
	*Older*	*20 (16%)*	*7 (11%)*	*53 (14%)*	*21 (13%)*	*1 (5%)*	*0.75*	
	*Oldest*	*4 (9%)*	*4 (15%)*	*34 (16%)*	*10 (13%)*	*0 (0%)*	*0.78*	
Signature 7	Total	15 (10%)	17 (25%)	98 (23%)	30 (16%)	5 (23%)	< 0.01*	UV light
	*Young*	*2 (12%)*	*1 (33%)*	*6 (16%)*	*2 (11%)*	*0 (0%)*	*0.75*	
	*Older*	*13 (10%)*	*16 (24%)*	*92 (24%)*	*28 (17%)*	*5 (24%)*	< 0.01*	
	*Oldest*	*4 (9%)*	*5 (19%)*	*51 (23%)*	*8 (11%)*	*2 (33%)*	0.03*	
Signature 8	Total	27 (18%)	11 (16%)	86 (20%)	39 (21%)	4 (18%)	0.90	Unknown
	*Young*	*5 (29%)*	*3 (100%)*	*5 (13%)*	*5 (28%)*	*0 (0%)*	0.02*	
	*Older*	*22 (17%)*	*8 (12%)*	*81 (21%)*	*34 (21%)*	*4 (19%)*	*0.51*	
	*Oldest*	*6 (13%)*	*3 (11%)*	*52 (24%)*	*17 (23%)*	*1 (17%)*	*0.38*	
Signature 9	Total	24 (16%)	9 (13%)	63 (15%)	34 (19%)	6 (27%)	0.38	Polymerase η
	*Young*	*2 (12%)*	*0 (0%)*	*6 (16%)*	*4 (22%)*	*0 (0%)*	*0.88*	
	*Older*	*22 (17%)*	*9 (14%)*	*57 (15%)*	*30 (18%)*	*6 (29%)*	*0.38*	
	*Oldest*	*9 (20%)*	*4 (15%)*	*35 (16%)*	*16 (21%)*	*0 (0%)*	*0.69*	
Signature 10	Total	5 (3%)	2 (3%)	25 (6%)	11 (6%)	3 (14%)	0.28	Defective polymerase POLE
	*Young*	*1 (6%)*	*0 (0%)*	*0 (0%)*	*0 (0%)*	*0 (0%)*	*0.27*	
	*Older*	*4 (3%)*	*2 (3%)*	*25 (6%)*	*11 (7%)*	*3 (14%)*	*0.20*	
	*Oldest*	*1 (2%)*	*2 (7%)*	*12 (5%)*	*4 (5%)*	*1 (17%)*	*0.48*	
Signature 11	Total	5 (3%)	4 (6%)	33 (8%)	13 (7%)	0 (0%)	0.34	Alkylating agents (e.g. temozolomide)
	*Young*	*2 (12%)*	*0 (0%)*	*3 (8%)*	*2 (11%)*	*0 (0%)*	*0.85*	
	*Older*	*3 (2%)*	*4 (6%)*	*30 (8%)*	*11 (7%)*	*0 (0%)*	*0.19*	
	*Oldest*	*0 (0%)*	*1 (4%)*	*16 (7%)*	*6 (8%)*	*0 (0%)*	*0.32*	
Signature 12	Total	10 (7%)	6 (9%)	59 (14%)	17 (9%)	3 (14%)	0.14	Unknown
	*Young*	*0 (0%)*	*0 (0%)*	*5 (13%)*	*1 (6%)*	*0 (0%)*	*0.53*	
	*Older*	*10 (8%)*	*6 (9%)*	*54 (14%)*	*16 (10%)*	*3 (14%)*	*0.31*	
	*Oldest*	*4 (9%)*	*2 (7%)*	*34 (16%)*	*8 (11%)*	*2 (33%)*	*0.29*	
Signature 13	Total	50 (34%)	42 (61%)	113 (26%)	46 (25%)	9 (41%)	< 0.01*	AID/APOBEC
	*Young*	*5 (29%)*	*2 (67%)*	*8 (21%)*	*2 (11%)*	*1 (100%)*	*0.07*	
	*Older*	*45 (35%)*	*40 (61%)*	*105 (27%)*	*44 (27%)*	*8 (38%)*	< 0.01*	
	*Oldest*	*16 (36%)*	*15 (56%)*	*57 (26%)*	*16 (21%)*	*3 (50%)*	< 0.01*	
Signature 14	Total	2 (1%)	0 (0%)	8 (2%)	9 (5%)	0 (0%)	0.13	Unknown
	*Young*	*0 (0%)*	*0 (0%)*	*4 (11%)*	*4 (22%)*	*0 (0%)*	*0.27*	
	*Older*	*2 (2%)*	*0 (0%)*	*4 (1%)*	*5 (3%)*	*0 (0%)*	*0.38*	
	*Oldest*	*0 (0%)*	*0 (0%)*	*1 (0%)*	*4 (5%)*	*0 (0%)*	*0.05*	
Signature 15	Total	18 (12%)	9 (13%)	74 (17%)	31 (17%)	3 (14%)	0.65	Defective DNA mismatch repair
	*Young*	*1 (6%)*	*0 (0%)*	*9 (24%)*	*2 (11%)*	*0 (0%)*	*0.43*	
	*Older*	*17 (13%)*	*9 (14%)*	*65 (17%)*	*29 (18%)*	*3 (14%)*	*0.84*	
	*Oldest*	*8 (18%)*	*4 (15%)*	*33 (15%)*	*15 (20%)*	*0 (0%)*	*0.77*	
Signature 16	Total	15 (10%)	9 (13%)	105 (24%)	37 (20%)	4 (18%)	< 0.01*	Unknown
	*Young*	*4 (24%)*	*0 (0%)*	*12 (32%)*	*2 (11%)*	*0 (0%)*	*0.48*	
	*Older*	*11 (9%)*	*9 (14%)*	*93 (24%)*	*35 (21%)*	*4 (19%)*	< 0.01*	
	*Oldest*	*4 (9%)*	*4 (15%)*	*48 (22%)*	*16 (21%)*	*1 (17%)*	*0.30*	
Signature 17	Total	2 (1%)	2 (3%)	8 (2%)	4 (2%)	0 (0%)	0.89	Unknown
	*Young*	*1 (6%)*	*0 (0%)*	*1 (3%)*	*0 (0%)*	*0 (0%)*	*0.53*	
	*Older*	*1 (1%)*	*2 (3%)*	*7 (2%)*	*4 (2%)*	*0 (0%)*	*0.71*	
	*Oldest*	*0 (0%)*	*0 (0%)*	*2 (1%)*	*3 (4%)*	*0 (0%)*	*0.23*	
Signature 18	Total	8 (5%)	2 (3%)	50 (12%)	14 (8%)	3 (14%)	0.03*	Unknown
	*Young*	*0 (0%)*	*0 (0%)*	*5 (13%)*	*1 (6%)*	*0 (0%)*	*0.53*	
	*Older*	*8 (6%)*	*2 (3%)*	*45 (12%)*	*13 (8%)*	*3 (14%)*	*0.08*	
	*Oldest*	*2 (4%)*	*1 (4%)*	*26 (12%)*	*4 (5%)*	*0 (0%)*	*0.28*	
Signature 19	Total	8 (5%)	4 (6%)	37 (9%)	14 (8%)	0 (0%)	0.55	Unknown
	*Young*	*1 (6%)*	*1 (33%)*	*1 (3%)*	*1 (6%)*	*0 (0%)*	*0.22*	
	*Older*	*7 (5%)*	*3 (5%)*	*36 (9%)*	*13 (8%)*	*0 (0%)*	*0.40*	
	*Oldest*	*4 (9%)*	*1 (4%)*	*21 (10%)*	*5 (7%)*	*0 (0%)*	*0.87*	
Signature 20	Total	8 (5%)	1 (1%)	13 (3%)	7 (4%)	1 (5%)	0.51	Defective DNA mismatch repair
	*Young*	*2 (12%)*	*0 (0%)*	*1 (3%)*	*0 (0%)*	*0 (0%)*	*0.31*	
	*Older*	*6 (5%)*	*1 (2%)*	*12 (3%)*	*7 (4%)*	*1 (5%)*	*0.66*	
	*Oldest*	*5 (11%)*	*1 (4%)*	*8 (4%)*	*4 (5%)*	*0 (0%)*	*0.30*	
Signature 21	Total	4 (3%)	1 (1%)	22 (5%)	5 (3%)	0 (0%)	0.46	Unknown
	*Young*	*0 (0%)*	*0 (0%)*	*3 (8%)*	*1 (6%)*	*0 (0%)*	*0.84*	
	*Older*	*4 (3%)*	*1 (2%)*	*19 (5%)*	*4 (2%)*	*0 (0%)*	*0.58*	
	*Oldest*	*3 (7%)*	*0 (0%)*	*10 (5%)*	*0 (0%)*	*0 (0%)*	*0.16*	
Signature 22	Total	3 (2%)	2 (3%)	47 (11%)	10 (5%)	2 (9%)	< 0.01*	Aristolochic acid
	*Young*	*0 (0%)*	*0 (0%)*	*6 (16%)*	*1 (6%)*	*0 (0%)*	*0.37*	
	*Older*	*3 (2%)*	*2 (3%)*	*41 (10%)*	*9 (5%)*	*2 (10%)*	< 0.01*	
	*Oldest*	*1 (2%)*	*1 (4%)*	*23 (11%)*	*2 (3%)*	*0 (0%)*	*0.11*	
Signature 23	Total	1 (1%)	1 (1%)	14 (3%)	3 (2%)	0 (0%)	0.44	Unknown
	*Young*	*0 (0%)*	*0 (0%)*	*0 (0%)*	*1 (6%)*	*0 (0%)*	*0.51*	
	*Older*	*1 (1%)*	*1 (2%)*	*14 (4%)*	*2 (1%)*	*0 (0%)*	*0.35*	
	*Oldest*	*0 (0%)*	*0 (0%)*	*7 (3%)*	*2 (3%)*	*0 (0%)*	*0.79*	
Signature 24	Total	17 (12%)	7 (10%)	55 (13%)	20 (11%)	4 (18%)	0.81	Aflatoxin
	*Young*	*7 (41%)*	*0 (0%)*	*3 (8%)*	*1 (6%)*	*0 (0%)*	0.02*	
	*Older*	*10 (8%)*	*7 (11%)*	*52 (13%)*	*19 (12%)*	*4 (19%)*	*0.38*	
	*Oldest*	*3 (7%)*	*3 (11%)*	*36 (16%)*	*6 (8%)*	*1 (17%)*	*0.21*	
Signature 25	Total	13 (9%)	4 (6%)	54 (13%)	12 (7%)	3 (14%)	0.11	Unknown
	*Young*	*1 (6%)*	*0 (0%)*	*3 (8%)*	*2 (11%)*	*0 (0%)*	*0.90*	
	*Older*	*12 (9%)*	*4 (6%)*	*51 (13%)*	*10 (6%)*	*3 (14%)*	*0.08*	
	*Oldest*	*3 (7%)*	*1 (4%)*	*35 (16%)*	*4 (5%)*	*0 (0%)*	0.05*	
Signature 26	Total	12 (8%)	2 (3%)	30 (7%)	12 (7%)	2 (9%)	0.63	Defective DNA mismatch repair
	*Young*	*0 (0%)*	*0 (0%)*	*1 (3%)*	*0 (0%)*	*0 (0%)*	*1.00*	
	*Older*	*12 (9%)*	*2 (3%)*	*29 (7%)*	*12 (7%)*	*2 (10%)*	*0.56*	
	*Oldest*	*7 (16%)*	*0 (0%)*	*14 (6%)*	*8 (11%)*	*0 (0%)*	*0.11*	
Signature 27	Total	1 (1%)	0 (0%)	6 (1%)	0 (0%)	2 (9%)	0.03*	Unknown
	*Young*	*0 (0%)*	*0 (0%)*	*0 (0%)*	*0 (0%)*	*0 (0%)*	*1.00*	
	*Older*	*1 (1%)*	*0 (0%)*	*6 (2%)*	*0 (0%)*	*2 (10%)*	0.03*	
	*Oldest*	*0 (0%)*	*0 (0%)*	*4 (2%)*	*0 (0%)*	*0 (0%)*	*0.84*	
Signature 28	Total	2 (1%)	2 (3%)	14 (3%)	2 (1%)	0 (0%)	0.48	Unknown
	*Young*	*0 (0%)*	*0 (0%)*	*0 (0%)*	*0 (0%)*	*0 (0%)*	*1.00*	
	*Older*	*2 (2%)*	*2 (3%)*	*14 (4%)*	*2 (1%)*	*0 (0%)*	*0.50*	
	*Oldest*	*0 (0%)*	*2 (7%)*	*6 (3%)*	*2 (3%)*	*0 (0%)*	*0.42*	
Signature 29	Total	19 (13%)	10 (14%)	92 (21%)	32 (18%)	4 (18%)	0.19	Chewing tobacco
	*Young*	*0 (0%)*	*1 (33%)*	*4 (11%)*	*6 (33%)*	*0 (0%)*	0.03*	
	*Older*	*19 (15%)*	*9 (14%)*	*88 (23%)*	*26 (16%)*	*4 (19%)*	*0.15*	
	*Oldest*	*8 (18%)*	*4 (15%)*	*46 (21%)*	*10 (13%)*	*3 (50%)*	*0.20*	
Signature 30	Total	18 (12%)	2 (3%)	58 (14%)	20 (11%)	4 (18%)	0.07	Unknown
	*Young*	*5 (29%)*	*1 (33%)*	*3 (8%)*	*0 (0%)*	*0 (0%)*	0.03*	
	*Older*	*13 (10%)*	*1 (2%)*	*55 (14%)*	*20 (12%)*	*4 (19%)*	0.01*	
	*Oldest*	*6 (13%)*	*0 (0%)*	*29 (13%)*	*11 (15%)*	*1 (17%)*	*0.22*	
Total	Total	146 (100%)	69 (100%)	429 (100%)	182 (100%)	22 (100%)		
	*Young*	*17 (100%)*	*3 (100%)*	*38 (100%)*	*18 (100%)*	*1 (100%)*		
	*Older*	*129 (100%)*	*66 (100%)*	*391 (100%)*	*164 (100%)*	*21 (100%)*		
	*Oldest*	*45 (100%)*	*27 (100%)*	*219 (100%)*	*75 (100%)*	*6 (100%)*		

Frequency and proportion of breast tumours of each PAM50 tumour type with each COSMIC signature present. Entries are number of samples with each signature present above a threshold of 6% (% of tumours of each PAM50 subtype). Signatures were identified using R package “deconstructSigs”. The *p*-values were calculated using a Fisher’s exact test (* indicates statistical significance at the 0.05 level). COSMIC: Catalogue Of Somatic Mutations In Cancer; HER2+: human epidermal growth factor receptor 2 over-expressing; PAM50: prediction analysis of microarrays 50 gene set

### Mutated genes

We found somatic tumour mutations including SNVs and small insertions and deletions within 46 pre-identified genes of interest [30]. The number of patients with at least one mutation in each gene for both the young (*n* = 91) and the older (*n* = 952) groups is reported in Table 3. One third of young-onset breast tumours contained at least one mutation in the *TP53* gene, making it the most commonly mutated gene among young breast cancer patients, followed by *PIK3CA* and *GATA3* mutations, each found in nearly one quarter of young cases. A significant difference in the proportion of young and older patients with mutations was found for five of the genes of interest. Of these, three were more commonly mutated among older patients (*PIK3CA*, *CDH1*, and *MAP3K1*), and two were more commonly mutated among young patients (*GATA3*, and *CTNNB1*).

**Table 3:** Mutated genes across age of onset groups

Gene	Young	Older	Oldest	p-value (young vs. older)	p-value (young vs. oldest)
*TP53*	31(34%)	320(33.6%)	133(28.6%)	0.91	0.32
*PIK3CA*	22(24%)	340(35.7%)	174(37.4%)	0.03*	0.02*
*GATA3*	20(22%)	123(12.9%)	50(10.8%)	0.02*	0.01*
*TAF1*	9(10%)	73(7.7%)	40(8.6%)	0.42	0.69
*PTEN*	7(8%)	58(6.1%)	32(6.9%)	0.50	0.82
*NF1*	6(7%)	97(10.2%)	57(12.3%)	0.36	0.15
*ATM*	6(7%)	70(7.4%)	35(7.5%)	1.00	1.00
*MORC4*	6(7%)	59(6.2%)	29(6.2%)	0.82	0.82
*ARID1A*	6(7%)	58(6.1%)	27(5.8%)	0.82	0.81
*NIPBL*	5(5%)	66(6.9%)	38(8.2%)	0.83	0.52
*CTNNB1*	5(5%)	14(1.5%)	10(2.2%)	0.02*	0.08
*CDH1*	4(4%)	170(17.9%)	96(20.6%)	0.0003*	0.0001*
*PIK3R1*	4(4%)	58(6.1%)	35(7.5%)	0.65	0.37
*MAP2K4*	4(4%)	53(5.6%)	26(5.6%)	0.81	0.80
*RB1*	4(4%)	49(5.1%)	23(4.9%)	1.00	1.00
*PPP2R1A*	4(4%)	34(3.6%)	23(4.9%)	0.57	1.00
*AKT1*	4(4%)	32(3.4%)	14(3%)	0.55	0.51
*MAP3K1*	3(3%)	108(11.3%)	66(14.2%)	0.01*	0.003*
*BRCA1*	3(3%)	47(4.9%)	26(5.6%)	0.61	0.60
*CHD4*	3(3%)	47(4.9%)	28(6%)	0.61	0.45
*EP300*	3(3%)	40(4.2%)	20(4.3%)	1.00	1.00
*HCFC2*	3(3%)	19(2%)	10(2.2%)	0.43	0.46
*RPL22*	3(3%)	13(1.4%)	9(1.9%)	0.16	0.43
*RUNX1*	2(2%)	62(6.5%)	33(7.1%)	0.11	0.10
*FBXW7*	2(2%)	33(3.5%)	16(3.4%)	0.76	0.75
*ZC3H13*	2(2%)	32(3.4%)	17(3.7%)	0.76	0.75
*RASA1*	2(2%)	31(3.3%)	16(3.4%)	1.00	0.75
*CTCF*	2(2%)	29(3%)	14(3%)	1.00	1.00
*ACVR2A*	2(2%)	28(2.9%)	17(3.7%)	1.00	0.75
*ARID5B*	2(2%)	26(2.7%)	18(3.9%)	1.00	0.76
*SPOP*	2(2%)	20(2.1%)	7(1.5%)	1.00	0.65
*RNF43*	2(2%)	14(1.5%)	11(2.4%)	0.64	1.00
*TBX3*	1(1%)	51(5.4%)	27(5.8%)	0.08	0.07
*CCAR1*	1(1%)	30(3.2%)	16(3.4%)	0.51	0.33
*CASP8*	1(1%)	26(2.7%)	14(3%)	0.50	0.48
*LARP7*	1(1%)	21(2.2%)	13(2.8%)	0.71	0.49
*CDKN1B*	1(1%)	13(1.4%)	6(1.3%)	1.00	1.00
*RPL5*	1(1%)	13(1.4%)	11(2.4%)	1.00	0.70
*KRAS*	1(1%)	12(1.3%)	8(1.7%)	1.00	1.00
*CD3G*	1(1%)	10(1.1%)	5(1.1%)	1.00	1.00
*LATS1*	1(1%)	10(1.1%)	6(1.3%)	1.00	1.00
*CCND1*	1(1%)	3(0.3%)	1(0.2%)	0.31	0.30
*PIK3R3*	0(0%)	18(1.9%)	12(2.6%)	0.39	0.23
*PRRG1*	0(0%)	13(1.4%)	10(2.2%)	0.62	0.38
*B2M*	0(0%)	8(0.8%)	4(0.9%)	1.00	1.00
*AP1S1*	0(0%)	7(0.7%)	5(1.1%)	1.00	1.00

Frequency and proportion of patients in each age of onset group with mutations in 46 genes of interest. Entries are number of samples with at least one mutation in a given gene (% of samples in age of onset group). The genes of interest were selected based on Berger et al.’s study of gynecologic and breast cancers using TCGA data [30]. The p-values were calculated using a chi squared test for difference in proportions (* indicates statistical significance at the 0.05 level). Age of onset groups were defined as “Young”: breast tumours diagnosed ≤40 years of age (n = 91), “Older”: breast tumours diagnosed > 40 years of age (n = 952), and “Oldest”: breast tumours diagnosed > 60 years of age (n = 465). HER2+: human epidermal growth factor receptor 2 over-expressing

When comparing gene mutations among young-onset cases and cases diagnosed after age 60 (*n* = 465), we found the same three genes (*PIK3CA*, *CDH1*, and *MAP3K1*) were significantly more commonly mutated in the oldest group than the young group. However, the only gene that was mutated in a larger proportion of the young group was *GATA3* (*p* = 0.01).

### Mutational load

**Fig. 1:**
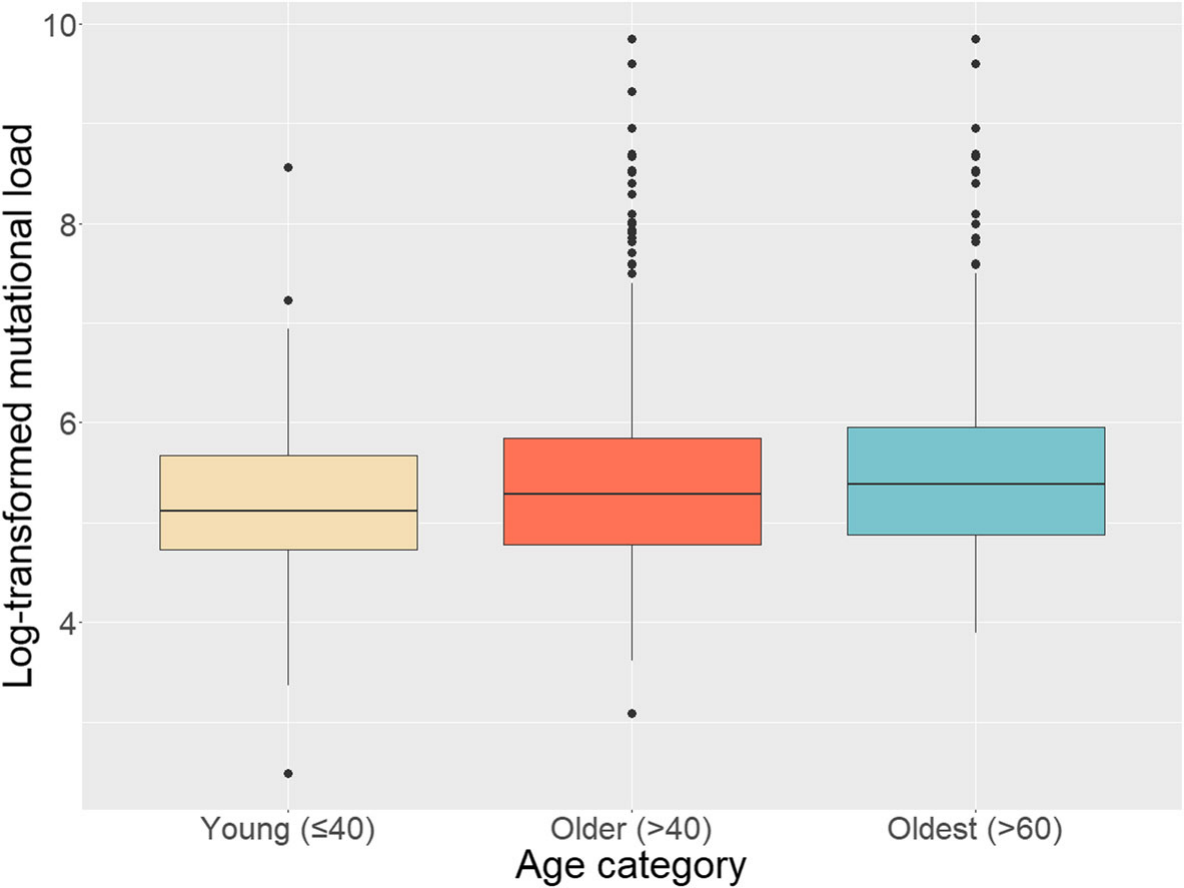
Log-transformed mutational load of young-onset breast tumours (diagnosed at ≤40 years of age, *n* = 91), older-onset breast tumours (diagnosed at > 40 years of age, *n* = 952), and oldest-onset breast tumours (the subset of the older group diagnosed at > 60 years of age, *n* = 465)

The median number of somatic mutations identified in the tumours of young breast cancer patients was 167 (IQR = 113–290.5), compared to 197.5 (IQR = 119–346) among older patients (Fig. 1). The distributions were positively skewed, so the data were log-transformed. A t-test of the log-transformed data showed marginal significance (*p* = 0.077). When comparing young-onset tumours to tumours diagnosed after age 60, the difference was statistically significant (*p* = 0.0057).

SNVs comprised on average 92.5% of all somatic mutations in our data. The median number of SNVs among young and older breast cancer patients was 159 (IQR = 105–268) and 181 (IQR = 109–323), respectively. The difference in number of SNVs between young- and older-onset tumours was statistically significant (*p* = 0.045). The *p*-value when comparing the young group to the over 60 age group was 0.0024.

### Mutation types

The proportion of SNVs represented by each mutation type was relatively similar between young- and older-onset groups (Table 4). For both groups, the most common SNVs were C > T (32 and 38% respectively), followed by C > G (both 17%) and C > A (17 and 16% respectively). Overall, there were fewer T > N mutations than C > N mutations (where N denotes any nucleotide). There was a significant difference (*p* < 0.05) between young and older tumours for two mutation types. Breast tumours from older patients had a higher proportion of C > T mutations (*p* = 0.009), but a lower proportion of C > A mutations (*p* = 0.015). These same trends were observed when comparing the young group to the > 60 age group. The variation in proportions of mutation types between samples are shown in Additional file 2: Figure S2. It also shows the overall higher proportion of C > N mutations, including some samples with over 90% C > N mutations.

**Table 4:** Mutation types across age of onset groups

Mutation Types	Young	Older	Oldest	TOTAL	p-value (young vs. older)	p-value (young vs. oldest)
C > A	7322 (17%)	103,484 (16%)	63,664 (17%)	110,806 (16%)	0.015*	0.01*
C > G	7012 (17%)	109,206 (17%)	60,090 (16%)	116,218 (17%)	0.59	0.11
C > T	13,500 (32%)	240,760 (38%)	149,492 (40%)	254,260 (38%)	0.009*	0.003*
T > A	4374 (10%)	50,176 (8%)	26,578 (7%)	54,550 (8%)	0.50	0.70
T > C	5764 (14%)	80,644 (13%)	47,058 (13%)	86,408 (13%)	0.68	0.41
T > G	3898 (9%)	50,366 (8%)	29,430 (8%)	54,264 (8%)	0.56	0.67

Frequency and proportion of mutation types among young-onset (diagnosed at ≤40 years of age, n = 89), older-onset (diagnosed at > 40 years of age, n = 949), and oldest-onset (diagnosed at > 60 years of age, n = 465) breast tumours. Entries are number of mutations (% of SNVs in age group). The p-values were calculated using a Welch Two Sample t-test (* indicates statistical significance at the 0.05 level). SNV: Single Nucleotide Variant

### Mutational signatures

#### deconstructSigs

The highest mean contributions among the young-onset group are attributable to signatures 3, 1, and 5, with contributions of 24.5, 14.1, and 5.6%, respectively (Additional file 3: Table S1). Sixteen signatures were found to have a mean contribution to mutational spectra of young-onset breast tumours over 1%. The only signature that was not identified in any of the young-onset samples was signature 27, whereas all 30 signatures were identified in some of the older-onset samples. The signatures with the highest contributions among older-onset tumours were signatures 3, 1, 2, 13, and 5, with mean contributions of 21.1, 16.3, 6.9, 5.8, and 5.4%, respectively. In the older age group, there were eighteen signatures with mean contributions over 1%. Contributions were slightly more heterogeneous among the older-onset group, with a greater variety of signatures making up the tumour mutations of this group. Within the oldest age group, the highest mean contributions came from signatures 3, 1, 2, and 5, with 19.5, 17.0, 7.0, and 6.4% contributions respectively. Seventeen signatures contributed over 1% of mutations overall in this age group, and all 30 signatures were detected. Sample-level contributions are shown in Additional file 4: Figure S3.

The signatures that were present in the greatest proportions among both the young- and older-onset groups were signature 1 (74 and 77.1%, respectively), and signature 3 (70 and 67.5%, respectively). Only signature 14 showed significant differences in prevalence between the young and older age groups, or between the young and oldest age groups, appearing in 9% of young cases, 2.2% of older cases, and 1.9% of the oldest cases.

#### SomaticSignatures

**Fig. 2:**
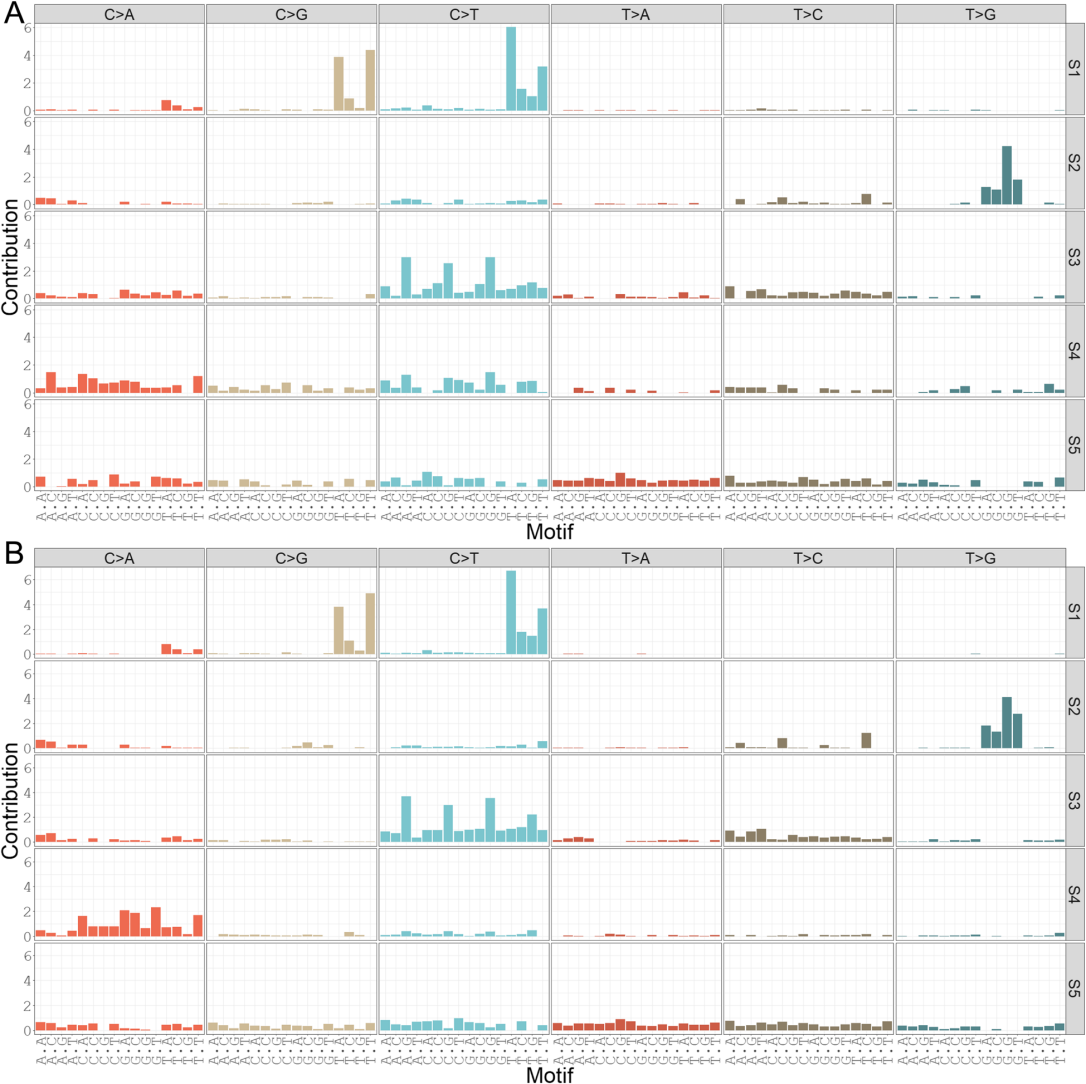
Mutational signatures among (A) young-onset breast tumours (diagnosed at ≤40 years of age, *n* = 89) and (B) older-onset breast tumours (diagnosed at > 40 years of age, *n* = 949) as identified using the R package “SomaticSignatures” (using NMF to identify five signatures). NMF: Non-negative Matrix Factorization

We used the “SomaticSignatures” package to decompose the mutations of breast tumours into five and sixteen mutational signatures for each of the young and older age groups. When decomposing mutations into five signatures, very similar patterns arose in each age group (Fig. 2). By visual inspection, the first signature S1 resembled a combination of signatures 2 and 13 from COSMIC [34]. Signature 2 had a higher frequency of C > T mutations whereas signature 13 had a higher frequency of C > G mutations. S2 resembled a non-validated signature “R1” observed previously by Alexandrov et al. and noted in their Supplementary Fig. 24 [35]. S3 resembled COSMIC signature 1. S4 and S5 lack peaks, making it difficult to discern their patterns with relatively few mutations. They could represent a combination of signatures that are present at low levels presenting with a flat profile, or may be noise.

When decomposed into sixteen signatures, recognizable signatures were more often divided into smaller components. In the young-onset group, S1 resembled COSMIC signatures 2 and 13 (Additional file 5: Figure S4). However, in the older group, S1 included C > T peaks resembling signature 2, and S2 has C > G peaks which are characteristic of signature 13 (Additional file 6: Figure S5). Contributions of these signatures are displayed in Additional file 7: Figure S6. S3 resembled the non-validated signature R1 mentioned previously. Similarly, in the older group, the R1 signature appears to be represented by S7. S4 in the young-onset and older-onset groups may represent signature 1 from COSMIC. The other signatures in the sixteen-signature decompositions did not have any strong peaks that match signatures in COSMIC, but may still represent mutational patterns that are parts of these signatures.

While in some cases, the mutational spectra were dominated by one or two signatures of the five-signature decomposition, most had contributions from at least three (Additional file 8: Figure S7). Tumours’ mutational spectra can be estimated by combining the mutational signatures with different contributions. These contributions for the young-onset group demonstrated that while some tumours’ mutations were comprised mainly of one mutational signature, most were comprised of multiple.

### Hierarchical clustering

**Fig. 3:**
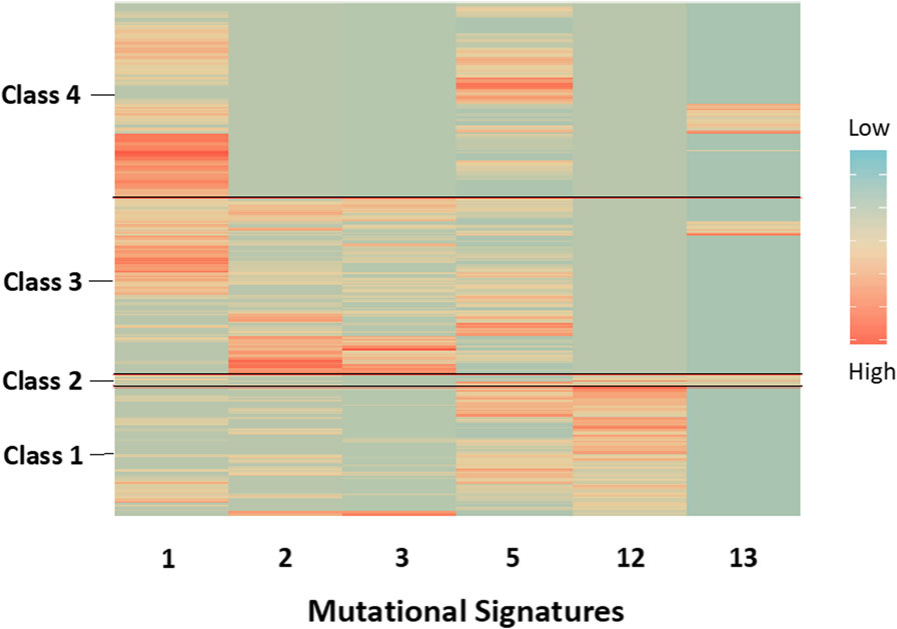
Recursively partitioned mixture model of the mutational signatures with the greatest variance using R package “RPMM”. Mutational signatures were identified using R package “deconstructSigs”, and signatures with the greatest variance were selected based on standard deviations and interquartile range

Examining individual signatures is limited, as it does not show the full picture when observing groups of tumours. We addressed this limitation by including a multi-signature grouping approach using “RPMM”. Six mutational signatures met our variability criteria with signatures 1, 2, 3, and 4 all having IQRs greater than 0% and SDs greater than 10%. Signature 12 had an SD of 4%, but an IQR greater than 0%, while signature 13 had an IQR of 0%, but a SD of 11%. The RPMM of these signatures resulted in four unique classes (Fig. 3). Class 1 (*n* = 213) included tumours with contributions from every signature except 13, but with the largest contributions from signatures 5 and 12. Class 2 (*n* = 20) consisted primarily of tumours with contributions from signatures 12 and 13. Class 3 (*n* = 292) included tumours with consistent contributions from signatures 1, 2, 3, and 5. Finally, Class 4 (*n* = 323) consisted of tumours with contributions from signatures 1 and 5, but no contributions from signatures 2, 3, and 12.

In our unadjusted analysis, there was evidence that PAM50 subtypes were differentially related to RPMM class membership (*p* < 0.001), but not for age (*p* = 0.41). In a multivariable analysis, young-onset cases were more likely to be in class 4 than any other class after adjusting for PAM50 subtype, but was not statistically significant (Odds Ratio (OR): 1.30, 95% CI: 0.81–2.10). Among subtypes, the HER2+ subtype was more likely to be in the class 3 than any other class when compared to basal-like tumours (OR: 2.53, 95% CI: 1.40–4.56).

## Discussion

We observed a difference in mutation prevalence among five genes of interest between young-onset breast tumours and those diagnosed among older patients. There was a higher overall number of somatic mutations in the tumours of older patients. Both age groups had more C > N than T > N mutations, but there was a significant difference in the proportions of C > T and C > A mutations by age. Analysis using “deconstructSigs” revealed that the prevalence of signature 14 was significantly higher among young-onset tumours. Signatures 1 and 3 were shown to be among the most common signatures in both age groups, both when examining prevalence and mean contributions. Decomposition of SNVs into novel mutational signatures by “SomaticSignatures” revealed patterns that resembled signatures 1, 2, and 13, and the non-validated signature R1 in both age groups. Analysis of trends relating to molecular subtype showed a difference by age for the basal-like subtype, and for some mutational signatures. When employing hierarchical clustering of the signatures with the most variability, we identified a unique combination of signatures that was significantly associated with HER2+ tumours.

Other studies have reported that young-onset patients are more likely to be diagnosed with more aggressive subtypes such as basal-like and HER2+. In our cohort, the basal-like subtype was more common among young-onset tumours, but this difference was only significant when comparing to tumours diagnosed after age 60. We did not find a significant difference in HER2+ tumours across age groups.

We observed the highest prevalence of somatic mutations among young breast cancer patients in the *TP53* (33%) and *PIK3CA* (24%) genes. Mutations in the *TP53* gene are associated with various types of cancer. A previous pan-cancer analysis using TCGA data by Campbell and Martinocorena reported prevalence of gene mutations among breast cancers without stratifying by age [36]. Their reported prevalence for *TP53* (33%), *PIK3CA* (34%), *GATA3* (10%), *MAP3K1* (7%), *CDH1* (7%), and *CTNNB1* (0%) were relatively similar to our findings, though they tended to be more similar to the proportions we observed in the older age group. This is unsurprising because of the much larger sample size of older patients in our sample.

We observed that a higher proportion of young patients had mutations in the *GATA3* (22% among young patients vs 12.9% among older patients and 10.8% among the oldest patients) and *CTNNB1* genes (5 and 1.5% among young and older patients, respectively), whereas the proportion was lower in the *PIK3CA*, *MAP3K1*, and *CDH1* genes compared to older patients. The high prevalence of *GATA3* mutations and particularly the higher prevalence among young patients motivates further investigation. Our findings are consistent with Azim et al., who observed a higher prevalence of *GATA3* mutations (15.2%) among young breast cancer patients (≤45 years), compared to < 10% in the intermediate and elderly age groups. The lower prevalence of *GATA3* mutations in their young age group may be due to the difference in age cutoff. Together, our results may suggest that *GATA3* mutations have a higher prevalence breast cancer patients younger than 40 years than those between 40 and 45 years. *GATA3* (GATA binding protein 3) encodes a transcription factor that is important in T-cell differentiation during immune response. It is particularly important in endothelial cells [37]. This may provide evidence for exposures involved in immunosuppression being involved in the etiology of some young-onset cases and that treatment may be able to be tailored to this pathway. *CTNNB1* (Catenin Beta 1) encodes a protein that is part of the Wnt pathway, and forms part of an adherens junction complex, responsible for intercellular structure and modulating cell growth. Mutations in this gene have been found to cause colorectal and ovarian cancers [38]. *CTNNB1* was found to be overexpressed in fulvestrant-resistant cell lines [39]. The Wnt pathway is strongly related to metabolic process, which may implicate obesity and diet as possible causes of young-onset breast cancer cases. However, this mutation has a low prevalence even among young-onset cases, and no difference was observed when comparing with the oldest age group, and thus caution should be taken when interpreting the difference with older-onset cases. *PIK3CA* is an oncogene that encodes a protein that is involved in the PI3K, AKT, and mTOR pathways [40]. *MAP3K1* encodes a kinase which acts as part of signal transduction cascades in the ERK, JNK kinase, NF-kappa-B pathways [41]. The higher proportion of older-onset tumours with mutations in these genes may support that these pathways are more often involved in the etiology of older-onset tumours while young-onset tumours are more likely to develop through the dysregulation of alternate stages or pathways. Mutations in the *CDH1* (Cadherin 1 gene) were also more common among older-onset breast tumours, and are related to increased proliferation, invasion, and metastasis of some cancer types, including breast cancer [42].

There was a significant difference in breast tumour mutational load between tumours diagnosed before age 40 and after age 60 which is driven by the higher number of SNVs among older patients. Although the small sample size of young patients means we should interpret this trend cautiously, it can be reasonably explained by a longer duration of lifestyle and environmental mutagen exposure for older patients. The most common types of SNV present in tumours across age groups were C > T, C > G, and C > A. We observed a higher proportion of C > A mutations among young-onset tumours. While this difference is statistically significant, the actual difference is small (17% vs 16%), so we suggest that it is not biologically meaningful. In contrast, we observed a significantly higher proportion of C > T mutations among older-onset tumours (38%) than young-onset tumours (32%)(*p* = 0.009). Liao et al. observed this same trend in their post-menopausal group compared to a pre-menopausal group, particularly at TCG contexts [13]. Depending on the mutation context, these C > T mutations may make up an important contribution to mutational signatures 1, 2, or 5 [34, 36]. Mutations of different types arise as a result of a combination of DNA damage and a (faulty) repair mechanism. Understanding the etiology of the mutations can direct prevention strategies, particularly if an exogenous mutagen is involved. Knowing which mutations are found in tumour cells may also be important for estimating prognosis and informing treatment.

We observed differences across PAM50 molecular subtypes for nine signatures, suggesting that the etiologic processes that contribute to these signatures also contribute to the development of particular tumour types. PAM50 has been shown to be valuable for predicting breast tumour progression, so mutational signatures may be important for prognosis. Our results showed that signatures 2 and 13 were significantly more prevalent among HER2+ breast tumours than other PAM50 subtypes. A study by Chen et al. observed that germline *APOBEC3A/B* deletion increases expression of isoform uc011aoc, which in breast cancer promotes mutational signatures 2 and 13 and immune response [43]. This research combined with our results suggest that isoform uc011aoc may be involved in the mutational profiles observed among HER2+ breast tumours. Signature 3 was more common among basal-like tumours, and is associated with mutations in the *BRCA1* and *BRCA2* genes and defective double-strand break repair by homologous recombination, suggesting a link between this erroneous repair mechanism and basal-like tumour development [34].

Signatures 1, 3, and 5 from COSMIC each had mean contributions over 5% among both age groups, and signatures 2 and 13 were also above this threshold in the older-onset group, showing higher heterogeneity of mutational signatures. Similarly, signatures 1 and 3 had the highest prevalence in both age groups. Signature 1 has been linked to an endogenous mutagenic process, and is thus more common in tumours diagnosed at later ages [34]. Alexandrov et al. who divided signature 1 into subtypes A and B, found that specifically in breast cancer signature 1B was correlated with age of diagnosis [35]. Signature 3 is linked to mutations in the *BRCA1* and *BRCA2* genes, but the etiology of signature 5 has yet to be elucidated [34]. Signatures 2 and 13 often co-occur, and are both attributed to mutagenic activity of cytidine deaminases from the AID/APOBEC family [34]. It has been suggested that this mutagenicity is triggered by retrotransposon jumping, viral infection, or tissue inflammation. The two different signatures arise via differential repair mechanisms [44].

Despite previous evidence that signature 1 was correlated with age [45], our findings do not show a significant difference in prevalence among young- and older-onset cases. A significant difference in prevalence between young- and older-onset groups was only seen in signature 14, appearing more frequently among young-onset tumours. Signature 14 was identified in fewer than 10% of tumours in both groups. COSMIC reports observing signature 14 in only five cases, including glioma and uterine cancer, and always alongside high mutational burdens of over 200 mutations/Mb. However, none of the samples in this study reached this mutation rate, suggesting that “deconstructSigs” identified signature 14 spuriously in these samples. Given the uncertainty of this finding, we cannot with confidence report any significant difference in the proportion of tumours with any COSMIC signature. Liao et al. saw a greater contribution from COSMIC signature 10 in their older age group [13]. While we did observe more contributions from signature 10 in the older-onset group, this difference was not statistically significant.

In both age groups, signatures resembling signatures 1, 2, 13, and R1 were observed using “SomaticSignatures”. The algorithm combined signatures 2 and 13 into a single signature when identifying five signatures, which may indicate high co-occurrence of signatures 2 and 13 among breast cancers. In previous research, signatures 2 and 13 are usually identified in the same samples [34]. They are attributed to activity of cytidine deaminases in the AID/APOBEC family [45]. The observation that multiple signatures contributed to most cases in the young group could indicate diverse mutagenic influences involved in tumour etiology. The results we found using “SomaticSignatures” and “deconstructSigs” yielded similar conclusions.

Specific tumours can harbor contributions from multiple mutational signatures and therefore it is important to determine if specific combinations are more prominent in young-onset cases. Analysis of single signatures does not provide a complete view of the complexities of somatic mutations within groups of tumours. In this study, however, we did not observe any combinations of mutational signatures that were significantly associated with young-onset cases. This lack of finding could be due to low statistical power to detect these combinations or a result of excessive heterogeneity in combinations of mutational signatures. While it was not statistically significant, the class that was most strongly related to young-onset cases had consistent contributions from signature 1 and 5. Alexandrov et al. found that signature 1B was correlated with age among 879 breast cancer patients [24], while the etiology of signature 5 remains unknown. In light of this, our findings could suggest that a subset of young-onset cases are more similar to older-onset breast cancer cases. In addition, signature 1 is associated with endogenous mutational processes, which could suggest a role for spontaneous mutations that may be due to other genetic factors.

Currently, screening women under age 50 without additional risk factors is not recommended in Canada. However, in light of the severe consequences of young-onset breast cancer, it is important to identify and treat cases in this population. The study of mutational signatures and other genomic markers among young-onset tumours can provide support for lifestyle or environmental exposures that increase risk of breast cancer. Uncovering the relevant risk factors using genomic epidemiology approaches could facilitate targeted screening programs for young women. This would increase early detection of young-onset tumours while reducing the potential harms of screening all young women. A greater understanding of lifestyle risk factors would also allow for prevention of breast cancer by recommending avoiding these exposures.

There are several strengths to our analysis. This analysis contributes to a limited number of studies that have included young-onset breast cancer cases. Our results provide information on the differences in mutational signatures found in breast tumours, in younger ages compared to older ages. No previous research that we know of has investigated the mutational signatures found in breast cancer stratified by age. The patterns in somatic mutations of breast tumours may be used to explain exposures related to young-onset breast cancer. Another strength of this analysis is that we examined multiple genomic features including mutation load, mutated genes, types of nucleotide changes and mutational signatures. Our study was able to identify specific combinations of mutational signature contributions that were related to young-onset breast cancer tumours. Understanding the role that genetics and biomarkers have in young-onset breast cancer may benefit future cancer prevention research and cancer control strategies.

There are some limitations to this analysis. Our study had a small sample size, specifically among the young-onset group which limits the power and generalizability of our analysis. We chose not to perform an adjustment for multiple comparisons when comparing gene mutations due to the exploratory nature of this study [46]. This should be taken into consideration when interpreting the results, and further studies should be carried out to confirm these findings. The data available through TCGA did not include occupational, lifestyle or environmental exposures such as BMI, and so we were unable to conduct analyses to link mutational signatures to potential etiologies among young-onset breast cancer cases. This limits our ability to link specific genomic mutations to various exposures which may have contributed to breast cancer. This analysis can only provide evidence that young-onset breast cancer cases have different mutational signatures when compared to breast cancer cases diagnosed over age 40. The reasons underlying this difference must be the topic of further investigations. This research is based on WES, thus we cannot draw conclusions related to mutations in stretches of the genome outside the exome. We did not investigate germline mutations or signaling pathways, and so did not produce new evidence linking mutational signatures to germline mutations or cellular signaling. Further research is underway to examine associations between exposures to mutational signatures and among young breast cancer patients.

Although our analysis using “SomaticSignatures” identified a signature in both age groups resembling R1 [35], this signature has not been validated. Further studies of the mutational signatures within breast tumours will be of interest in determining whether R1 is reproducible in other samples. It may be of interest for subsequent studies to look further into signature 14 to either confirm or reject our finding of a higher prevalence among young-onset breast tumours. The etiology of signature 14 remains unknown, so further investigation would be needed to determine the mechanism that results in this pattern of mutations.

Both breast cancer and its treatments can have severe consequences for young patients. Mutations caused by radiation therapy have the possibility of initiating recurrent tumour development. The potential life span of young-onset patients results in a longer period of surveillance [14, 47]. Premature menopause may be triggered by adjuvant treatments such as chemotherapy and may lead to premature loss of fertility, while hormone suppression therapy (i.e. tamoxifen) can lead to menopausal symptoms and long term loss of bone mineral density [2]. Negative psychosocial effects such as anxiety, depressive symptoms, and lower energy were observed to be worse among younger breast cancer survivors [2, 48, 49]. Larger studies with proportionally more young-onset samples are required to fully characterize breast cancers among this age group. Future studies should also include information on lifestyle and environmental exposures, which could allow for the elucidation of common exposures and molecular features among these patients to inform prevention policies.

## Conclusion

The incidence of young-onset breast cancer has been increasing. Characterization of genomic features of these cancers could provide etiologic clues and possible biomarkers that inform prognosis and treatment. The results of this exploratory study provide some evidence that the mutational landscape among young-onset cases is different from older-onset patients. Larger studies with data on medical history and lifestyle factors are required to clarify these relationships.

## Supplementary information

**Additional file 1: Figure S1**. Contribution of 30 COSMIC mutational signatures by PAM50 subtype. Contributions of 30 COSMIC mutational signatures to the mutational spectra of (A) young-onset breast tumours (diagnosed at ≤40 years of age, *n* = 77) and (B) older-onset breast tumours (diagnosed at > 40 years of age, *n* = 771). Sorted by PAM50 subtype. Cases with unknown subtype were excluded. HER2+: human epidermal growth factor receptor 2 over-expressing; PAM50: prediction analysis of microarrays 50 gene set.

**Additional file 2: Figure S2**. Proportion of mutation types. Proportions of the six mutation types among SNVs observed in (A) young-onset breast tumours (diagnosed at ≤40 years of age, *n* = 89) and (B) older-onset breast tumours (diagnosed at > 40 years of age, *n* = 949), sorted by percent of C > T mutations. SNV: Single Nucleotide Variant.

**Additional file 3: Table S1.** Signature contributions across age of onset groups. Summary of contributions of COSMIC signatures to mutational spectra, including the mean contribution of each signature in each age of onset group, and the prevalence in each age of onset group.

**Additional file 4: Figure S3**. Contributions of 30 COSMIC mutational signatures. Contributions of 30 COSMIC mutational signatures to the mutational spectra of (A) young-onset breast tumours (diagnosed at ≤40 years of age, *n* = 89) and (B) older-onset breast tumours (diagnosed at > 40 years of age, *n* = 949). Sorted by signature contribution.

**Additional file 5: Figure S4**. Sixteen mutational signatures among young-onset tumours. Mutational signatures among young-onset (n = 89) breast tumours as identified using the R package “SomaticSignatures” (using NMF to identify sixteen signatures). NMF: Non-negative Matrix Factorization.

**Additional file 6: Figure S5**. Sixteen mutational signatures among older-onset tumours. Mutational signatures among older-onset (n = 949) breast tumours as identified using the R package “SomaticSignatures” (using NMF to identify sixteen signatures). NMF: Non-negative Matrix Factorization.

**Additional file 7: Figure S6**. Contributions of sixteen mutational signatures. Contribution of sixteen signatures identified using R package “SomaticSignatures” to the mutational spectra of (A) young-onset breast tumours (n = 89) and (B) older-onset breast tumours (n = 949).

**Additional file 8: Figure S7**. Contributions of five mutational signatures. Contributions of five signatures identified using R package “SomaticSignatures” to the mutational spectra of (A) young-onset breast tumours (diagnosed at ≤40 years of age, n = 89) and (B) older-onset breast tumours (diagnosed at > 40 years of age, n = 949).

## Data Availability

The results published here are in whole or part based upon data generated by The Cancer Genome Atlas managed by the NCI and NHGRI. Information about TCGA can be found at http://cancergenome.nih.gov. In particular, the variant calls and clinical data analyzed during the current study are available through the Genomic Data Commons Data Portal, dbGaP accession number = phs000178, https://portal.gdc.cancer.gov/projects/TCGA-BRCA. The PAM50 subtype data are available from cBioPortal, https://www.cbioportal.org/study/summary?id=brca_tcga_pub [22].

## Abbreviations

COSMIC: Catalogue Of Somatic Mutations In Cancer
HER2+: Human epidermal growth factor receptor 2 over-expressing
HR: Hazard ratio
IQR: Interquartile range
NMF: Non-negative matrix factorization
OR: Odds ratio
PAM50: Prediction analysis of microarrays 50 gene set
RPMM: Recursively partitioned mixture model
SNV: Single nucleotide variant
TCGA: The Cancer Genome Atlas
TNBC: Triple-negative breast cancer
VCF: Variant call format
WES: Whole exome sequencing
