# Supplementary figures and images for: Mutational landscape differences between young-onset and older-onset breast cancer patients
Source: BMC Cancer. 2020 Mar 12;20:212. doi: 10.1186/s12885-020-6684-z (PMC7068998; doi:10.1186/s12885-020-6684-z)

## Slide 1
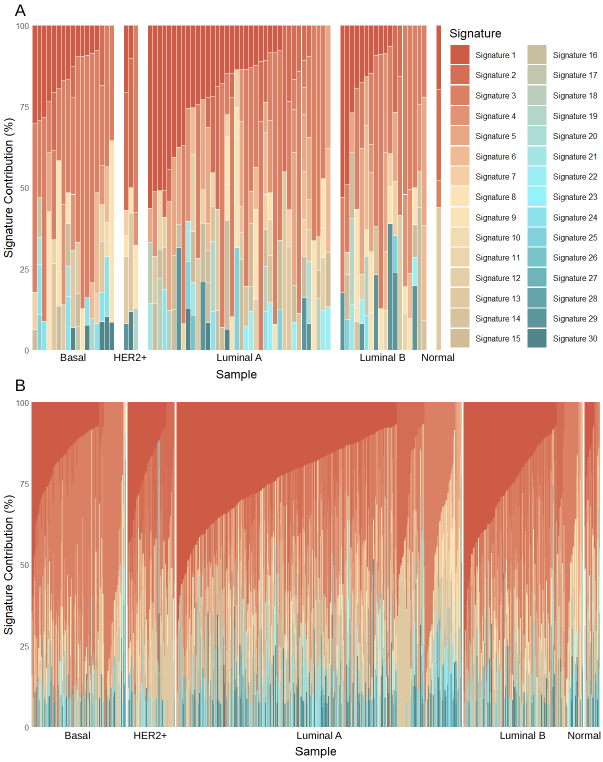

A
Basal
HER2+
Luminal A
Luminal B
Normal
B
Basal
HER2+
Luminal A
Luminal B
Normal

Supplement: Supplementary file 1 — Additional file 1: Figure S1. Contribution of 30 COSMIC mutational signatures by PAM50 subtype. Contributions of 30 COSMIC mutational signatures to the mutational spectra of (A) young-onset breast tumours (diagnosed at ≤40 years of age, n = 77) and (B) older-onset breast tumours (diagnosed at > 40 years of age, n = 771). Sorted by PAM50 subtype. Cases with unknown subtype were excluded. HER2+: human epidermal growth factor receptor 2 over-expressing; PAM50: prediction analysis of microarrays 50 gene set. [file 12885_2020_6684_MOESM1_ESM.pptx]

## Slide 1
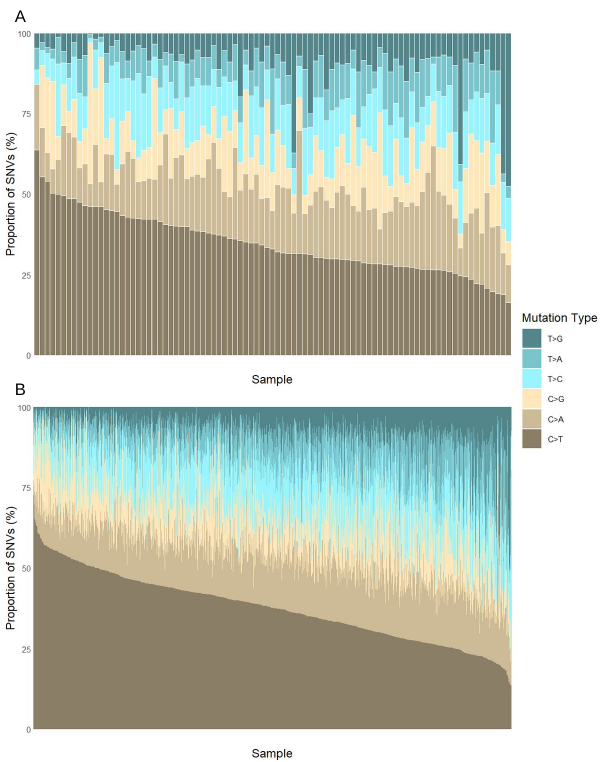

A
B

Supplement: Supplementary file 2 — Additional file 2: Figure S2. Proportion of mutation types. Proportions of the six mutation types among SNVs observed in (A) young-onset breast tumours (diagnosed at ≤40 years of age, n = 89) and (B) older-onset breast tumours (diagnosed at > 40 years of age, n = 949), sorted by percent of C > T mutations. SNV: Single Nucleotide Variant. [file 12885_2020_6684_MOESM2_ESM.pptx]

## Slide 1
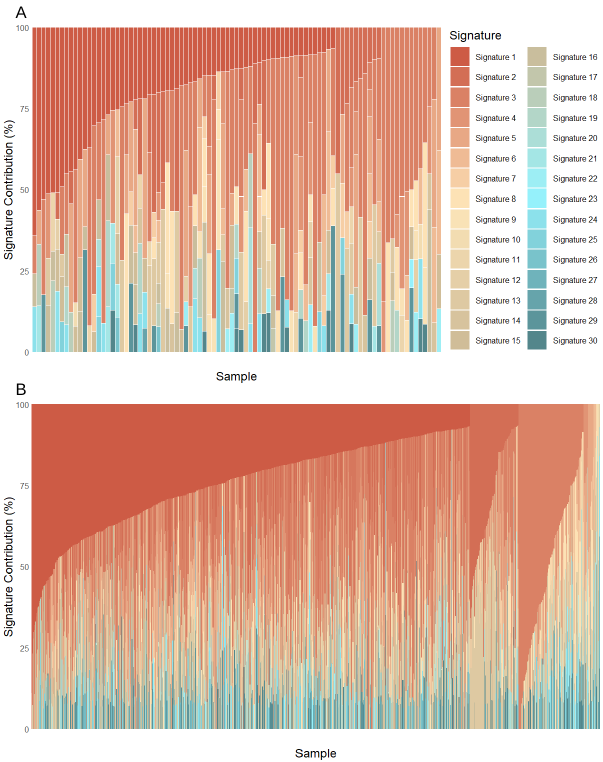

A
B

Supplement: Supplementary file 4 — Additional file 4: Figure S3. Contributions of 30 COSMIC mutational signatures. Contributions of 30 COSMIC mutational signatures to the mutational spectra of (A) young-onset breast tumours (diagnosed at ≤40 years of age, n = 89) and (B) older-onset breast tumours (diagnosed at > 40 years of age, n = 949). Sorted by signature contribution. [file 12885_2020_6684_MOESM4_ESM.pptx]

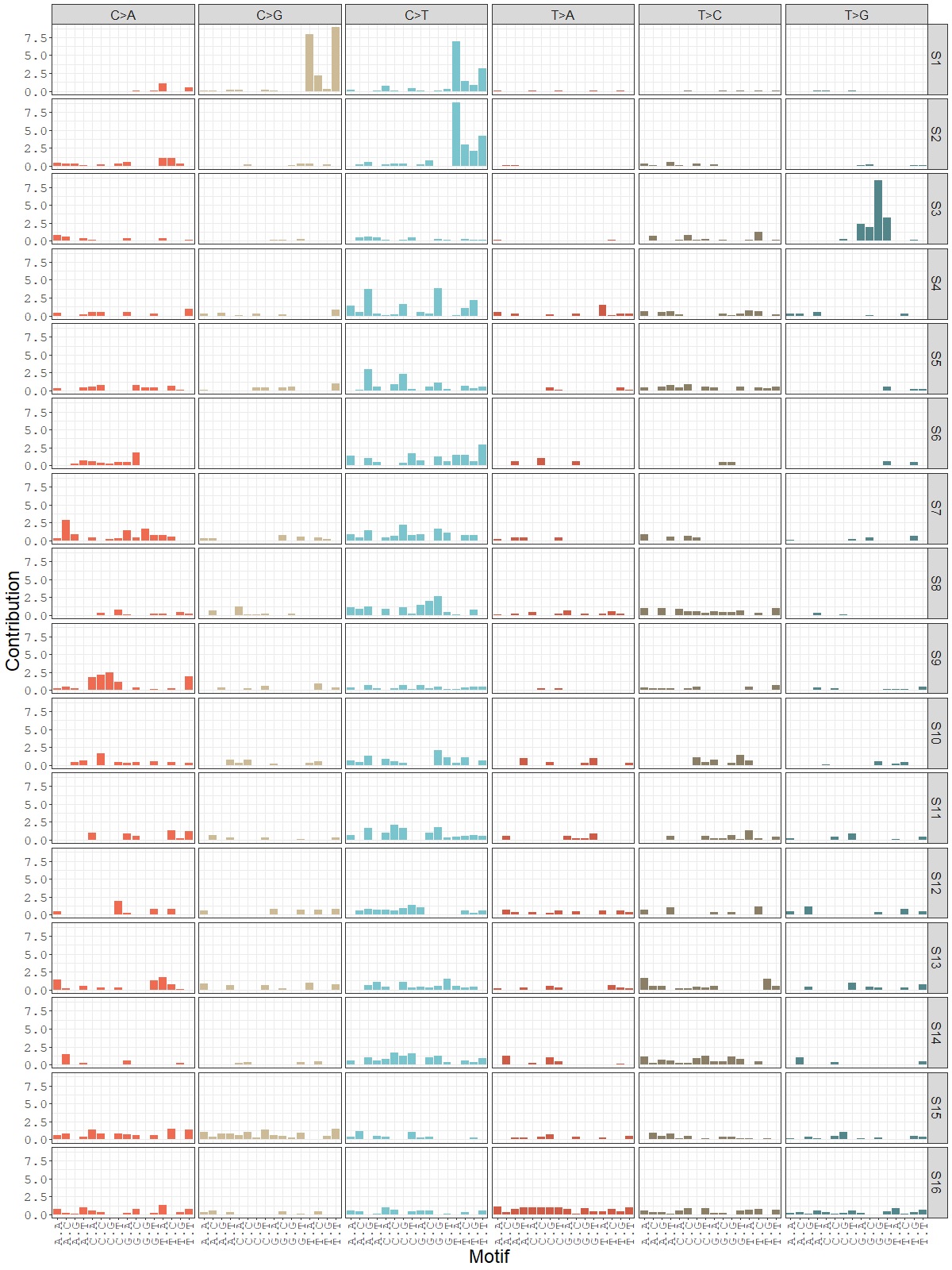

Supplement: Supplementary file 5 — Additional file 5: Figure S4. Sixteen mutational signatures among young-onset tumours. Mutational signatures among young-onset (n = 89) breast tumours as identified using the R package “SomaticSignatures” (using NMF to identify sixteen signatures). NMF: Non-negative Matrix Factorization. [file 12885_2020_6684_MOESM5_ESM.png]

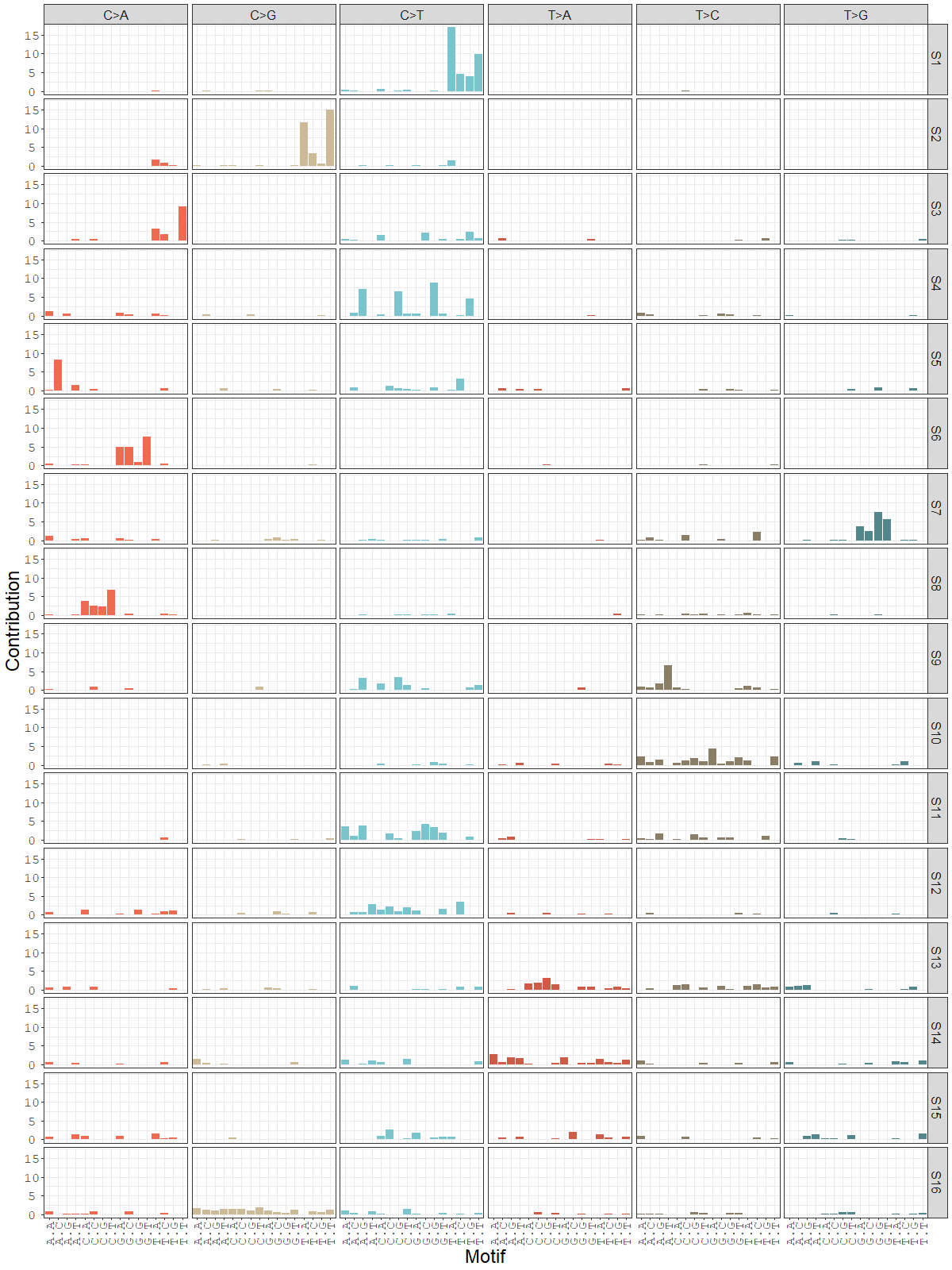

Supplement: Supplementary file 6 — Additional file 6: Figure S5. Sixteen mutational signatures among older-onset tumours. Mutational signatures among older-onset (n = 949) breast tumours as identified using the R package “SomaticSignatures” (using NMF to identify sixteen signatures). NMF: Non-negative Matrix Factorization. [file 12885_2020_6684_MOESM6_ESM.png]

**A**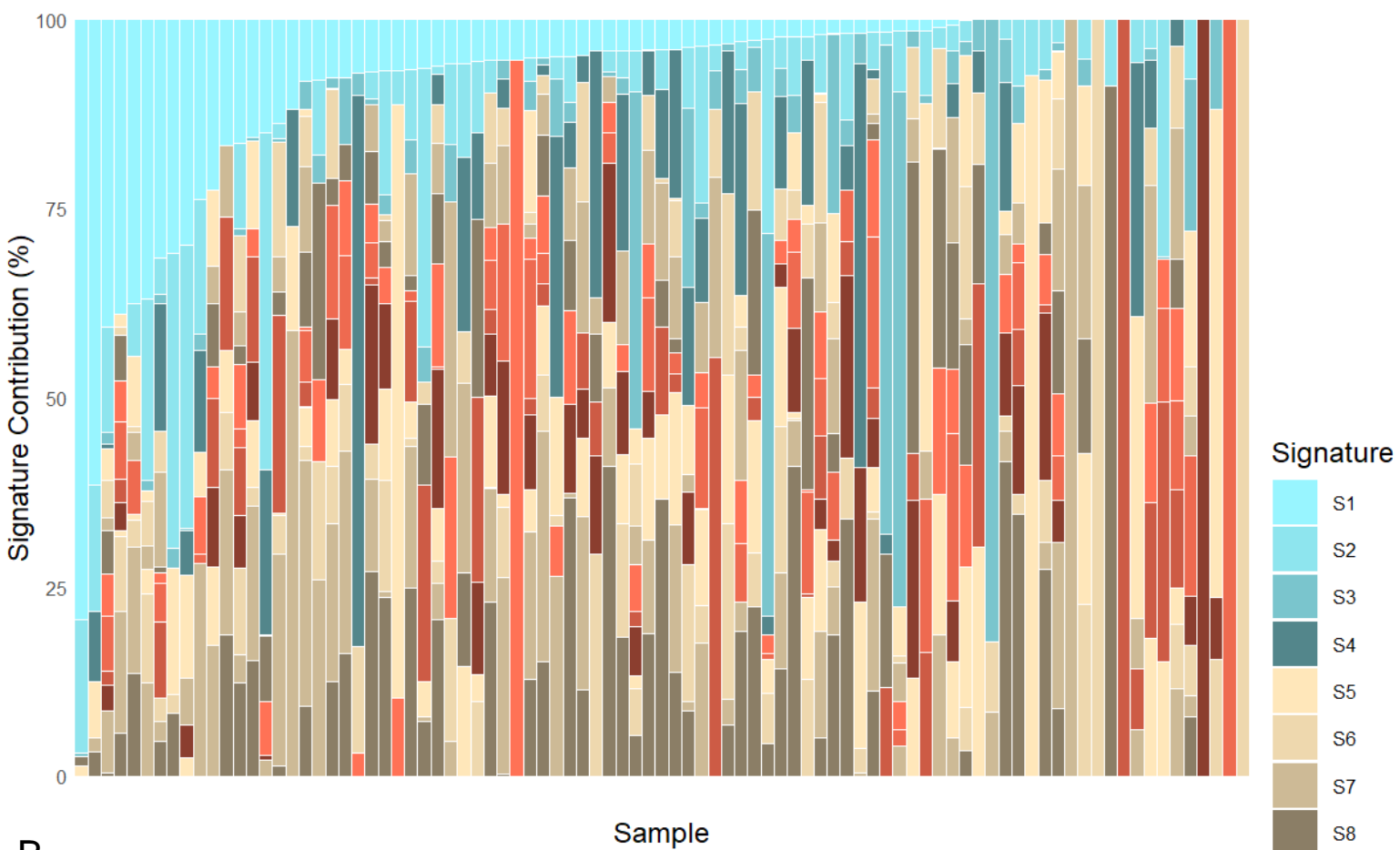**B**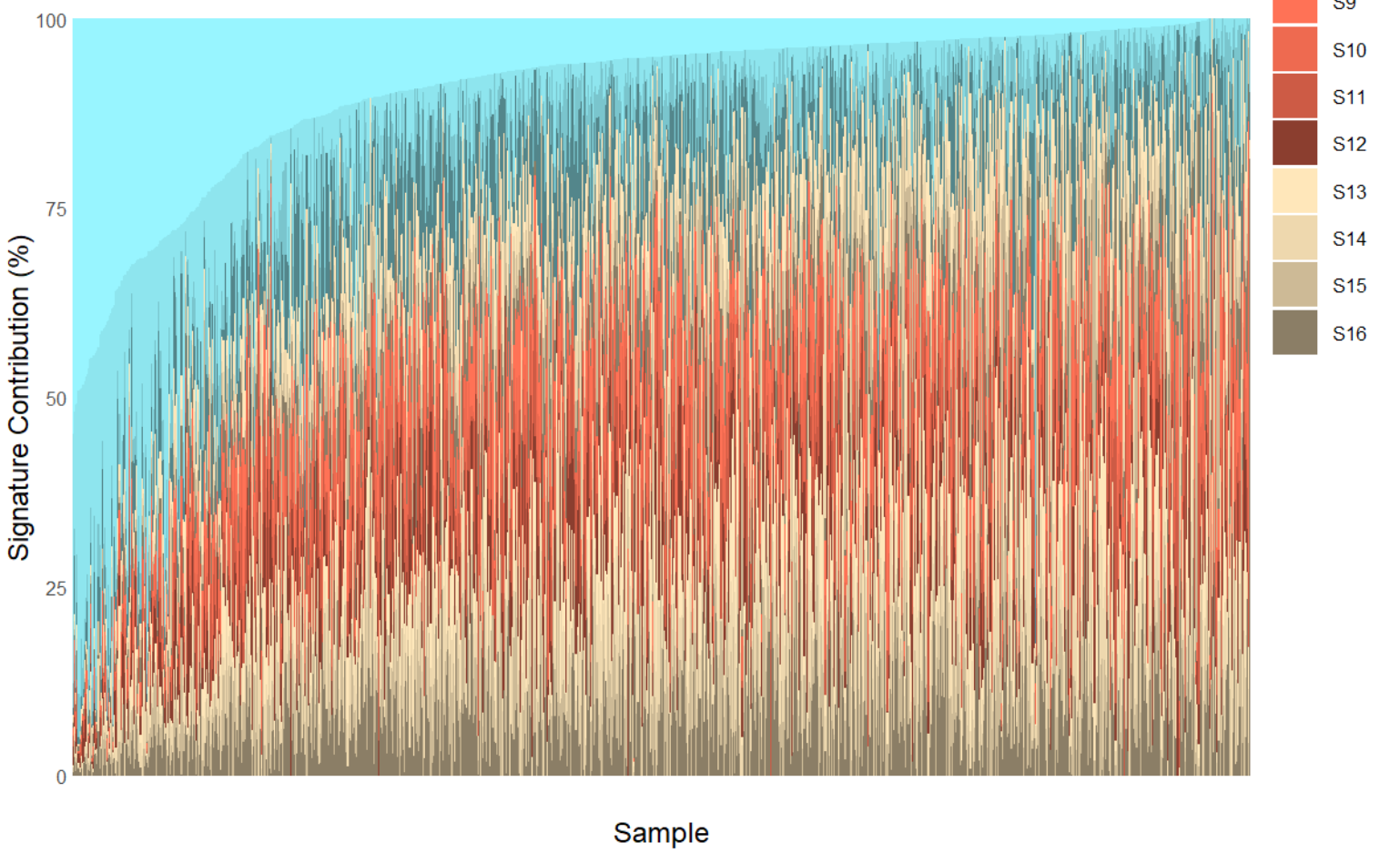

Supplement: Supplementary file 7 — Additional file 7: Figure S6. Contributions of sixteen mutational signatures. Contribution of sixteen signatures identified using R package “SomaticSignatures” to the mutational spectra of (A) young-onset breast tumours (n = 89) and (B) older-onset breast tumours (n = 949). [file 12885_2020_6684_MOESM7_ESM.pdf]

## Slide 1
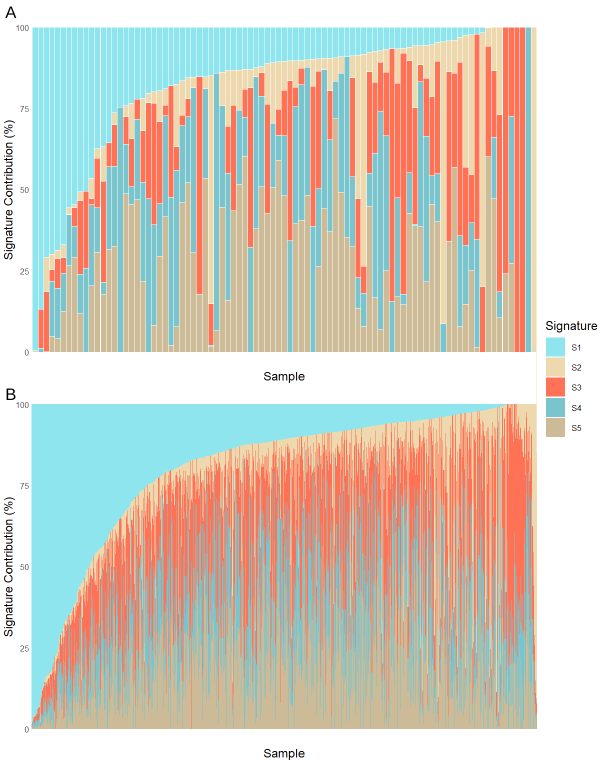

A
B

Supplement: Supplementary file 8 — Additional file 8: Figure S7. Contributions of five mutational signatures. Contributions of five signatures identified using R package “SomaticSignatures” to the mutational spectra of (A) young-onset breast tumours (diagnosed at ≤40 years of age, n = 89) and (B) older-onset breast tumours (diagnosed at > 40 years of age, n = 949). [file 12885_2020_6684_MOESM8_ESM.pptx]
