# Supplementary material for: Mutational landscape differences between young-onset and older-onset breast cancer patients
Source: BMC Cancer. 2020 Mar 12;20:212. doi: 10.1186/s12885-020-6684-z (PMC7068998; doi:10.1186/s12885-020-6684-z)
Supplement: Supplementary file 3 — Additional file 3: Table S1. Signature contributions across age of onset groups. Summary of contributions of COSMIC signatures to mutational spectra, including the mean contribution of each signature in each age of onset group, and the prevalence in each age of onset group. [file 12885_2020_6684_MOESM3_ESM.docx]

Signature contributions across age of onset groups

|  | **Mean Contribution** | | | **Proportion of cases with signature present** | | | **p-value (young vs. older)** | **p-value (young vs. oldest)** |
| --- | --- | --- | --- | --- | --- | --- | --- | --- |
|  | *Young* | *Older* | *Oldest* | *Young* | *Older* | *Oldest* |  |  |
| Signature 1 | 14.1% | 16.3% | 17.0% | 74% | 77.1% | 79.4% | 0.856 | 0.669 |
| Signature 2 | 4.7% | 6.9% | 7.0% | 28% | 36.7% | 37.0% | 0.595 | 0.649 |
| Signature 3 | 24.5% | 21.0% | 19.5% | 70% | 67.4% | 65.2% | 0.893 | 0.727 |
| Signature 4 | 3.2% | 1.7% | 1.7% | 20% | 12.6% | 12.7% | 0.595 | 0.649 |
| Signature 5 | 5.6% | 5.4% | 6.4% | 26% | 27.4% | 31.6% | 0.911 | 0.669 |
| Signature 6 | 3.0% | 2.2% | 2.4% | 19% | 15.0% | 15.5% | 0.813 | 0.727 |
| Signature 7 | 1.2% | 2.0% | 2.0% | 13% | 20.3% | 19.4% | 0.595 | 0.669 |
| Signature 8 | 3.6% | 3.4% | 3.6% | 22% | 19.5% | 22.2% | 0.856 | 0.947 |
| Signature 9 | 1.6% | 1.6% | 1.8% | 16% | 15.1% | 16.3% | 0.991 | 0.916 |
| Signature 10 | 0.2% | 0.6% | 0.7% | 2% | 5.4% | 4.9% | 0.735 | 0.669 |
| Signature 11 | 0.7% | 0.5% | 0.5% | 8% | 6.2% | 6.2% | 0.856 | 0.774 |
| Signature 12 | 0.9% | 1.3% | 1.5% | 8% | 11.3% | 13.1% | 0.813 | 0.669 |
| Signature 13 | 4.7% | 5.8% | 5.0% | 22% | 31.6% | 28.8% | 0.595 | 0.669 |
| Signature 14 | 0.8% | 0.2% | 0.2% | 9% | 2.2% | 1.9% | 0.006* | 0.012* |
| Signature 15 | 1.8% | 1.9% | 1.9% | 17% | 17.3% | 17.6% | 0.991 | 0.916 |
| Signature 16 | 3.2% | 3.2% | 3.3% | 24% | 19.7% | 20.2% | 0.816 | 0.732 |
| Signature 17 | 0.2% | 0.2% | 0.1% | 2% | 1.8% | 1.3% | 0.911 | 0.732 |
| Signature 18 | 1.2% | 1.1% | 1.0% | 9% | 9.0% | 8.2% | 0.992 | 0.910 |
| Signature 19 | 1.0% | 0.9% | 0.9% | 8% | 7.6% | 8.6% | 0.991 | 0.910 |
| Signature 20 | 0.6% | 0.5% | 0.5% | 4% | 4.5% | 5.2% | 0.992 | 0.910 |
| Signature 21 | 0.7% | 0.3% | 0.2% | 7% | 3.6% | 3.0% | 0.595 | 0.649 |
| Signature 22 | 0.8% | 0.6% | 0.7% | 9% | 7.4% | 7.5% | 0.872 | 0.831 |
| Signature 23 | 0.1% | 0.2% | 0.2% | 1% | 2.4% | 2.6% | 0.816 | 0.727 |
| Signature 24 | 1.8% | 1.7% | 1.6% | 17% | 12.3% | 12.7% | 0.735 | 0.669 |
| Signature 25 | 0.9% | 1.2% | 1.2% | 8% | 9.5% | 10.5% | 0.880 | 0.732 |
| Signature 26 | 0.2% | 0.7% | 0.7% | 2% | 6.4% | 6.7% | 0.595 | 0.649 |
| Signature 27 | 0.0% | 0.1% | 0.1% | 0% | 0.9% | 0.9% | 0.816 | 0.727 |
| Signature 28 | 0.1% | 0.2% | 0.2% | 1% | 2.5% | 2.2% | 0.816 | 0.749 |
| Signature 29 | 2.3% | 2.6% | 2.6% | 15% | 19.6% | 19.6% | 0.758 | 0.669 |
| Signature 30 | 1.1% | 1.5% | 1.6% | 13% | 12.0% | 12.5% | 0.893 | 0.910 |

Summary of contributions of COSMIC signatures to mutational spectra, including the mean contribution of each signature across all tumours in each age of onset group, and the proportion of cases in each age of onset group where the signature was present above a threshold of 6%. The p-values were calculated using a chi-square test for differences in proportions of cases where each signature was present, and were adjusting for the false discovery rate (* indicates statistical significance at the 0.05 level). Age of onset groups were defined as “Young”: breast tumours diagnosed at ≤40 years of age (n=89), “Older”: breast tumours diagnosed >40 years of age (n=949), and “Oldest”: breast tumours diagnosed >60 years of age (n=465).
